# Tetramethylammonium Cation: Directionality and Covalency in Its Interactions with Halide Ions

**DOI:** 10.1021/acs.inorgchem.2c00600

**Published:** 2022-06-06

**Authors:** Diego M. Gil, Jorge Echeverría, Santiago Alvarez

**Affiliations:** Departament de Química Inorgànica i Orgànica and Institut de Química Teòrica i Computacional IQTC-UB, Universitat de Barcelona, Martí i Franquès 1-11, 08028 Barcelona, Spain

## Abstract

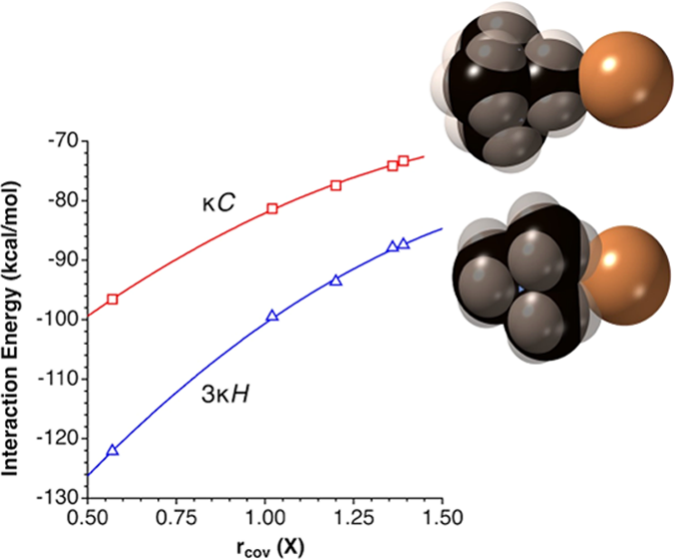

The degree of interpenetration of the van der Waals crusts of two atoms, represented by a penetration index, is defined to better quantify the meaning of the nonbonding contact distances between two atoms, which should allow us to compare different atom pairs on the same footing. The structural trends of the intermolecular contacts between the tetramethylammonium cation (TMA) and halogen atoms are reviewed, and a computational study of model X···TMA ion pairs (X = F, Cl, Br, I, Au) is presented. The results disclose two energy minima, in each of which the anion simultaneously interacts with three hydrogen atoms. The bonding mechanisms in the two cases are discussed based on the results of the tools of the trade that provide a consistent picture in which a distribution of charges significantly varies not only around each different atom but is also strongly dependent on the distance to the central N atom. This behavior, together with some non-negligible covalent character of the interionic interaction, is not predicted from a single-molecular electrostatic potential map of the TMA cation.

## Abstract 2

Two interaction topologies sustain interactions between the tetramethylammonium cation and halide (or auride) anions. A simple dependence of the interaction energy on the anion’s atomic radius is found, as a result of a refined combination of ionic (major component), covalent, and dispersion forces, in which the interpenetration of the van der Waals crusts of the interacting atoms seems to have a role.

## Introduction

Our interest in interactions of tetramethylammonium (TMA) and related alkylammonium cations with halides arises because, while an important electrostatic component should be expected in the formation of ion pairs or multiple anion–cation interactions in the solid state, the single positive charge is distributed among the four methyl groups, possibly making covalent and dispersion contributions non-negligible. In fact, such interactions could be classified as weak or nonconventional hydrogen bonds, formed by a weak hydrogen donor and a strong hydrogen acceptor.^1^

Understanding and harnessing the forces that bond *n*-methylammonium cations to anions are of importance in at least two fields of chemistry. On the one hand, wide research has been conducted in the last few decades in an attempt to design encapsulating organic cations as hosts of anions, referred to as “ammonium-based anionic receptors,”^2,3^ although it has focused mainly on the R_3_N-H^+^ group to form hydrogen bonds with anions, where R_3_ represents three branches of an encapsulating polycyclic compound. An interesting case is the design of a macrocyclic receptor that recognizes ion pairs formed by a methylammonium cation and a halide.^4^

On the other hand, the quaternary ammonium cations (QACs) present in quaternary ammonium salts (QAS) have been known to act as biocides and disinfectants for more than one century^5^ and have still today an astounding number of applications in detergents, shampoos, cosmetics, artificial tears, household products, and the popular hydrogels for hand sanitation. Almost half (46.4%) of the 580 disinfectants considered by the US Environmental Protection Agency as effective against COVID-19 contain a QAS in its formulation. The most common QAS contains one benzyl group, a long even-numbered straight alkyl chain, and two methyl groups (**1**), referred to generically as benzalkonium chlorides (BCs), and an example of a new generation of QAS is didecyldimethylammonium chloride (DDAC, **2**, *n* = 4).
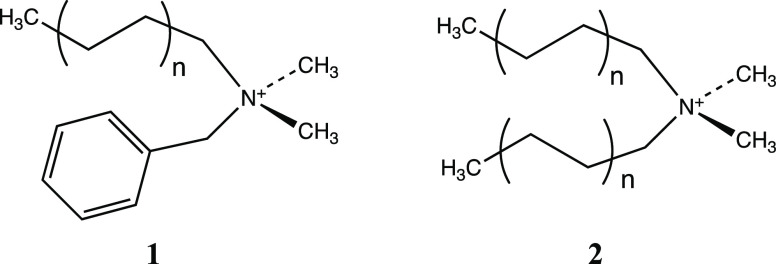


The Gemini QACs are designed by connecting two conventional QAC units through a bridge, resulting in a dication with a slightly different design, with two hydrophilic heads and two hydrophobic tails (**3**).^6^ In general, the mode of action of QACs plausibly takes advantage of their amphiphilic character, binding anionic species such as phospholipids through the dimethylammonium functions, and to the hydrophobic core of the plasma membrane via the alkyl chains.

Recently also, cetyltrimethylammonium bromide (CTAB) has been shown to self-assemble to produce micelles that give rise to the formation of uniform intracrystalline mesoporosity in zeolites. The mesoporous materials thus created present enhanced activity as fuel cracking catalysts.^7^
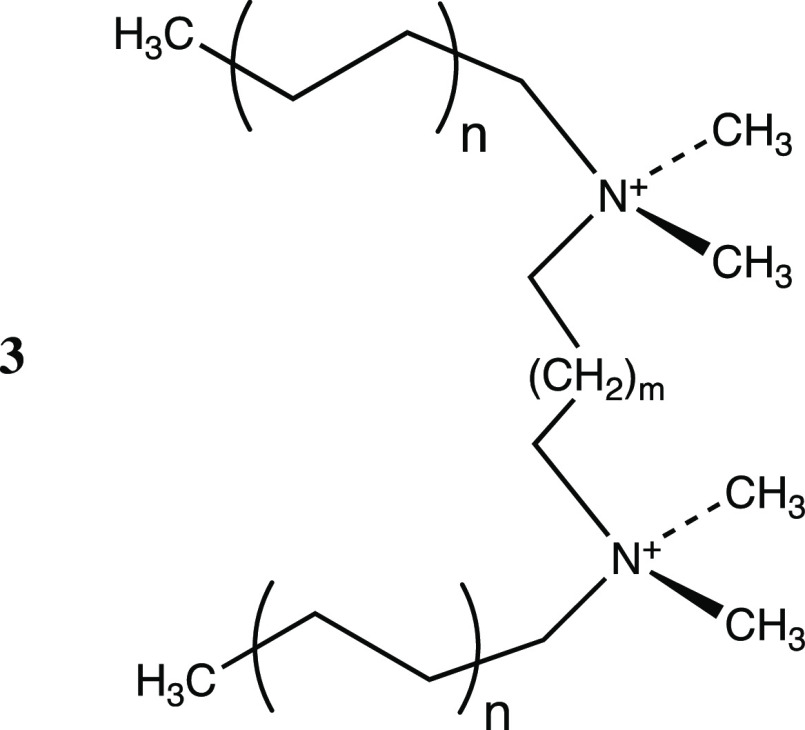


### Binding Modes of *n*-Methylammonium to Anions

**Figure 1:**
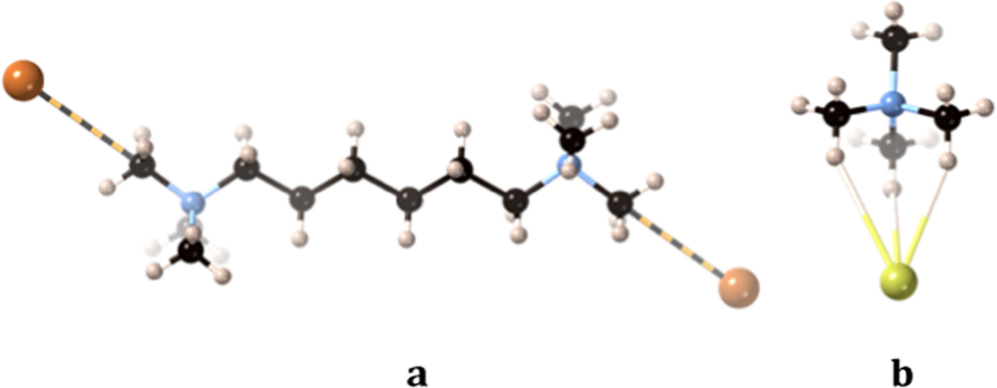
Partial view of (**a**) the κ^3^H (or κ^1^C) interaction between bromide anions and the methylammonium groups in the crystal structure of [Me_3_N(CH_2_)_6_NMe_3_]I_2_·H_2_O^20^ and (**b**) the 3κ^1^H contact in (NMe_4_)Cl·(HOPhCOOH)_2_·H_2_O.^21^

A structural analysis of contacts between n-methylammonium cations (*n* = 1–4) and halide anions, carried out by us in the CSD and ICSD structural databases, shows that they present a variety of topologies schematically represented in **4**–**9**, as well as intermediate situations. To distinguish those topologies, we name them using the kappa convention established by the IUPAC to indicate through which donor atom (or atoms) is a particular ligand coordinated to a metal.^8^ Thus, binding of a methyl group to an anion through *n* atoms is represented by the Greek letter kappa with *n* as a superindex, followed by the symbol(s) of the interacting atom(s), e.g., κ^1^H (**4**). An ambiguity appears for the topology with the anion at the same distances to the three hydrogen atoms of the same methyl group (Figure 1a) that could be considered as κ^3^H (**6**) or κ^1^C (**7**), an issue to be discussed in this paper. For simplicity, however, we will consistently use the κ^1^C notation that will be shown to be in better agreement with the nature of the interaction. In many instances, an anion is linked to one H atom of two (**8**) or three (**9**) different methyl groups of the same ammonium cation, a situation that is indicated by a numeric prefix and the atomic symbols of the linked atoms, e.g., 3κ^1^H,H′,H″, which will be shortened throughout this paper to 3κ^1^H, given the equivalence of the three H atoms. Examples of the binding topologies that can be found in several simple *n*-methylammonium salts are presented in Table 1.
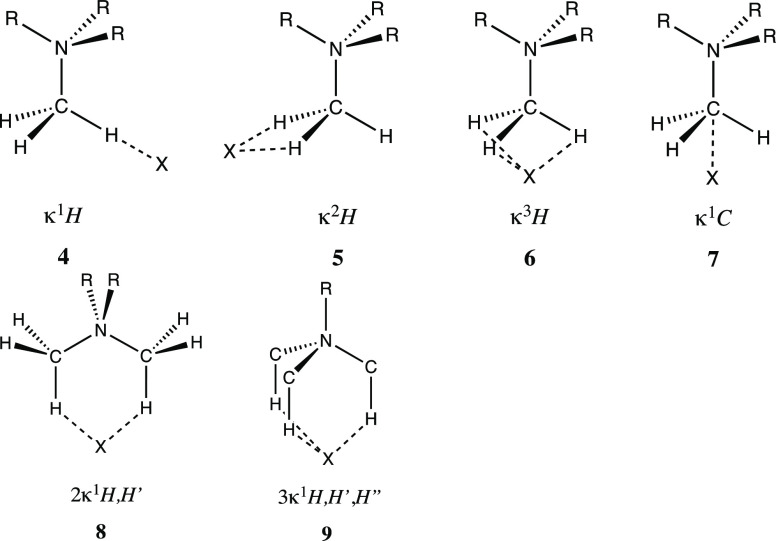


**Table 1:** Modes of Bonding of Simple Methylammonium Cations to Halide Anions

compound	topology	ref
(H_3_NMe)F	κ^1^H	(12)
[H_3_C(CH_2_)_13_NMe_3_]Br	κ^2^H	(13)
[MeH_2_N-CH_2_-CH_2_-NHMe_2_]Cl	κ^3^H or κ^1^C	(14)
(H_2_NMe_2_)Br	2κ^1^H	(15)
(HNMe_3_)Br	3κ^1^H	(16)
(HNMe_3_)Cl(HCl_2_)	κ^1^H, κ^2^H, 2κ^1^H	(17)
(HNMe_3_)I	2κ^1^H, 3κ^1^H	(18)
(HNMe_3_)Br	2κ^1^H, 3κ^1^H	(19)

**Figure 2:**
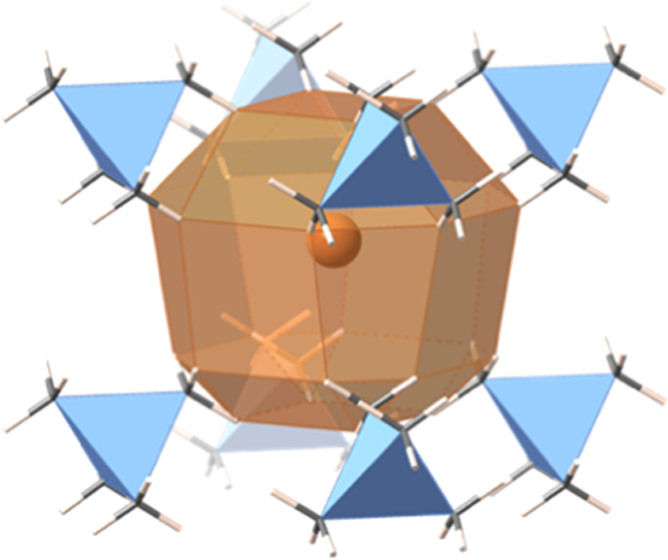
Coordination sphere around the bromide anion in the crystal structure of NMe_4_Br.^11^ Each tetrahedron is a TMA cation.

Two attractive examples of the sophisticated supramolecular architectures that can be assembled around a single TMA cation, highlighted earlier by one of us,^9^ are the TMA auride^10^ and the isostructural bromide,^11^ whose polyhedral environment of hydrogen atoms is shown in Figure 1b. The anion is linked by 24 hydrogen atoms forming an asymmetric rhombicuboc-tahedron (Figure 2). In these compounds, the asymmetry of the Archimedean polyhedron arises because each of the four nearest-neighbor cations in the upper part of Figure 2 presents a bonding-mode intermediate between κ^1^ and 3κ^1^, reflected in the case of the bromide by one short (2.98 Å) and two long (3.36 Å) Br···H distances, while the four cations at the lower, narrower part have contact topologies intermediate between κ^2^ and κ^3^, each cation having two Br···H distances of 3.48 Å and one distance of 3.58 Å. The same topological pattern is found in the structure of the TMA auride.^10^

### Interpenetration of van der Waals Crusts in Atom–Atom Interactions

When dealing with noncovalent interactions, we must take into account that the frontiers between an A···B pair of nonbonded and weakly bonded atoms, generally marked by the sum of their van der Waals radii *v*_A_ and *v*_B_, are fuzzy. To make it clear, let us recall that the van der Waals radii are statistical constructs deduced from an approximately Gaussian distribution of experimental distances distinctly longer than the sum of covalent radii *r*_A_ and *r*_B_. It has been stressed that noncovalent interactions may appear roughly within ±0.7 Å of the van der Waals radii sum.^12^ A similar reflection applies to the covalent bond distances since the covalent radii have standard deviations that range from 0.01 to 0.12 Å.^12^

**Figure 3:**
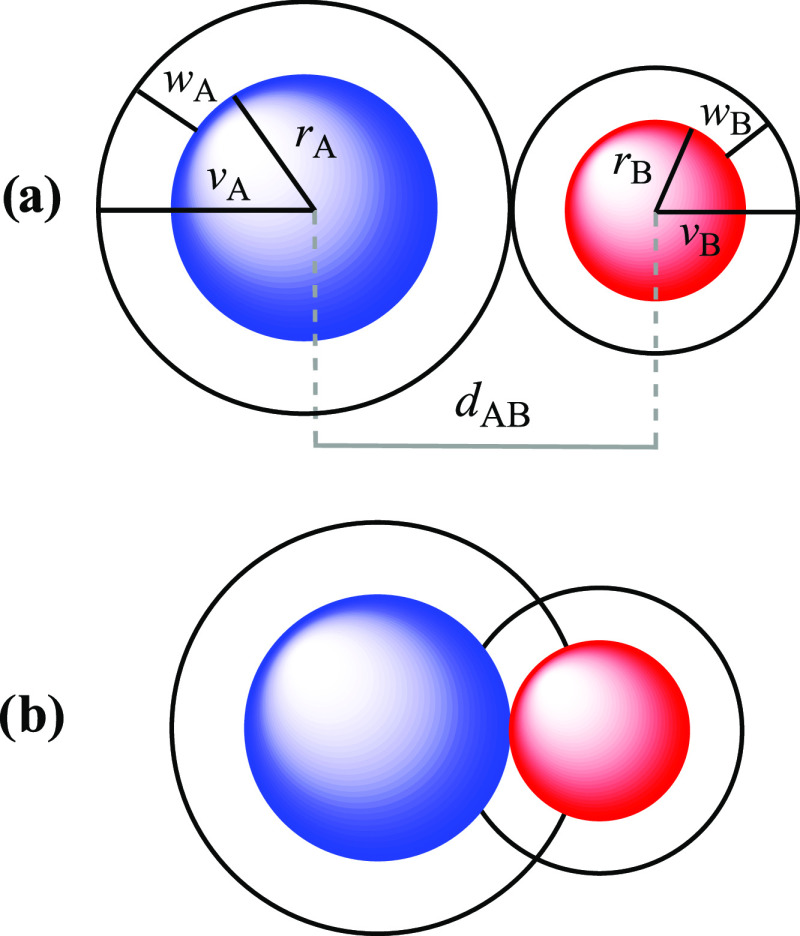
(a) Definition of the van der Waals crusts of widths *w*_A_ and *w*_B_ for atoms A and B from the covalent and van der Waals radii (*r* and *v*, respectively), and visual description of the case of two contacting van der Waals spheres with no interpenetration (*p*_AB_ = 0%). (b) Schematic description of the case in which the two atoms are at a distance equal to the sum of the covalent radii (*d*_AB_ = *r*_A_ + *r*_B_) that corresponds to a penetration index *d*_AB_ = 100%.

To evaluate the relevance of an intermolecular A···B contact, usually, the interatomic distance *d*_AB_ is compared with the sum of the van der Waals radii, but to compare different contacts, often a corrected or “normalized” distance is used, simply defined as the difference between the van der Waals sum and the distance. As an alternative parameter, Lommerse and co-workers^16^ proposed the quotient of the distance over the van der Waals sum, λ = *d*_AB_/(*ν*_A_ + *ν*_B_) that adopts values smaller than 1 for shorter, and larger than 1 for longer, distances than the van der Waals sum. We think that it is more informative to compare the contact distance with both the covalent and van der Waals radii sums. Our reasoning goes as follows (Figure 3). We first define the van der Waals crust of an atom A as the space comprised between a sphere of radius *v*_A_ (van der Waals radius) and a sphere of radius *r*_A_ (covalent radius) that encloses most of its valence electron density. The van der Waals crust has therefore a width *w*_A_ = *v*_A_ – *r*_A_. Then, we consider that two atoms A and B at a distance *d*_AB_, at which the interpenetration between their van der Waals crust can vary between 0 (for *d*_AB_ = *v*_A_ + *v*_B_) and 100% (for *d*_AB_ = *r*_A_ + *r*_B_), the two canonical situations which we would conventionally define as a van der Waals contact and a chemical bond, respectively. Thus, to convert an interatomic distance in a percentage of interpenetration of the van der Waals cores, we need only divide the difference between the van der Waals sum and the distance by the sum of the widths of the two crusts, as done in eq 1, in which we define a penetration index *p*_AB_ that indicates the percentage of interpenetration of the van der Waals crusts of A and B from 0% (canonical van der Waals contact) to 100% (canonical bond distance). A preliminary exploration of the various types of noncovalent interactions indicates that this might be a useful tool to classify them according to the levels of penetration they can achieve.
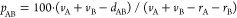
1

Note that a negative value of *p*_AB_ indicates that the fuzzy van der Waals spheres are conventionally not interpenetrated, although weak attractive interactions cannot be ruled out. The absolute value of a negative penetration index provides a qualitative description of the interaction as probably weakly attracting (small negative values) or as noninteracting or repulsive (large negative values). Conversely, values of *p*_AB_ larger than 100% indicate covalent bonding and provide a qualitative indication of the strength of the bond, including a differentiation of single and multiple bonds, as will be shown in a forthcoming publication. In summary, the values of the penetration index are intended to provide semiquantitative information about interactions within the van der Waals crust regions and should be used only as a qualitative indicator beyond the limits of 0 and 100%. A preliminary exploration of the various types of noncovalent interactions indicates that this might be a useful tool to classify them according to the levels of penetration they can achieve.

### Penetration Indices for the Methylammonium–Halide Contacts

**Figure 4:**
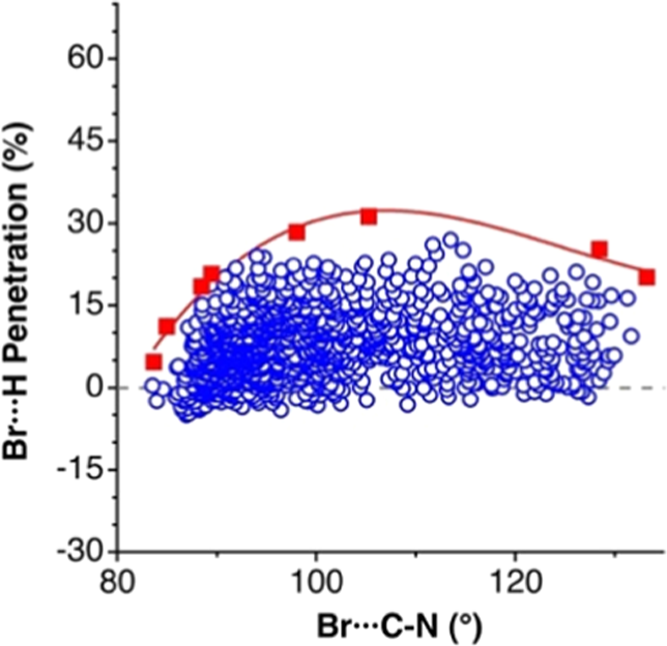
Distribution of the penetration indices for κ^1^H···Br contacts in trialkylammonium bromides as a function of the Br···C–N angle (circles), with the highest penetrations singled out as squares, and the solid line obtained by least-squares fitting providing a simplified view.

**Figure 5:**
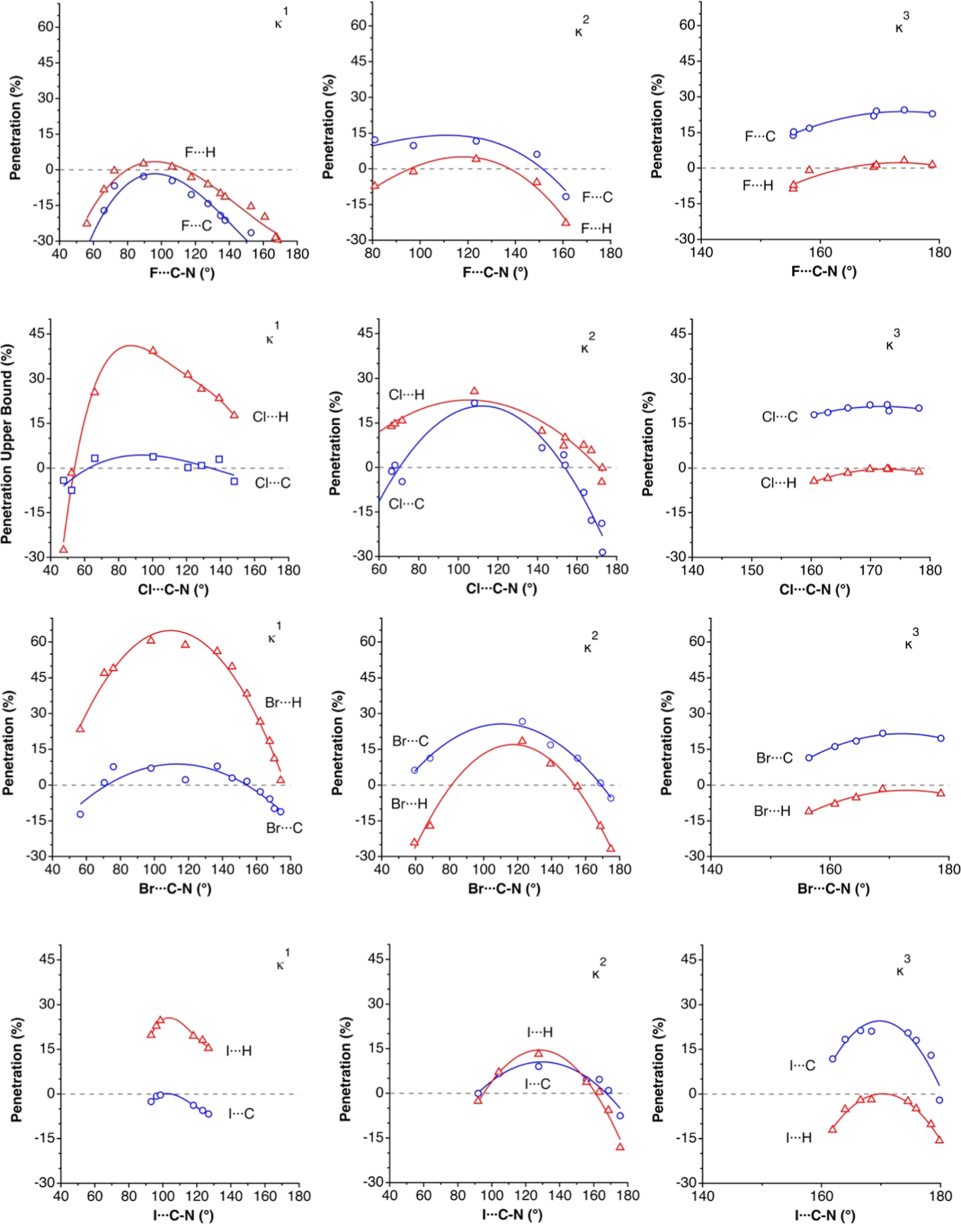
Maximum penetration curves for halide···methylammonium interactions of κ^1^ (left column), κ^2^ (middle column), and κ^3^ (right column) contacts with H (triangles) and C (squares) atoms. Contacts of each halide are shown in the same row, from fluoride (top row) to iodide (bottom row).

In this section, we analyze the penetration of the van der Waals crusts of *n*-methylammonium cations and halide anions considering both the X···H and X···C contacts of the methyl groups. Since in many crystal structures one finds more than one of the bonding modes **4**–**9**, as well as intermediate situations, we have carried out searches in the CSD^22^ for contact distances of up to 3.6 Å (X···H) and 3.8 Å (X···C), imposing restrictions on the X···C–N and/or X···H–C angles for κ^1^H (**4**, **8** or **9**), κ^2^H (**5**), and κ^3^H (**6** or **7**) contact topologies. From the representation of the distances as a function of the X···C–N angle, we extract the points that represent approximately the highest penetration for an interval of that angle and a least-squares fitting provides us with a simplified view of the whole set of compounds. This procedure is illustrated in Figure 4 for the case of the κ^1^-H···Br contacts. Such a simplified view of the X···H and X···C contacts between halides and methyl groups of *n*-methylammonium cations is presented in Figure 5, now covering a wider range of angles and controlling the topology not only by the angle but also by the existence of only one (κ^1^), two (κ^2^), or three (κ^3^) contacts, respectively.

The analysis of the penetration plots of Figure 5 allows us to establish some clear trends:

(1) The penetration of the hydrogen crust in the κ^1^H contacts is higher for all of the halides than that of the carbon atom, an unsurprising result given the restriction to nearly linear X···H–C units in the structural searches.
(2) The greatest penetration indices among κ^1^ contacts appears for all halides at an X···C–N angle of 110°, i.e., the tetrahedral angle that corresponds to a linear X···H–C arrangement.
(3) While there is practically no interpenetration of the van der Waals crusts of F and H in the κ^1^H topology, penetrations of up to 60% are seen for heavier halides.
(4) Different from κ^1^H systems, the κ^3^H ones present higher penetrations of X with the C than with the H atoms, reaching penetration indices of up to 25 and 3%, respectively. This suggests that it would be more adequate to term those interactions κ^1^C, as will be done from here on.
(5) The κ^2^H binding presents similar penetrations (up to 25%) of the halide with carbon and hydrogen atoms, thus suggesting that the interaction is significantly delocalized over both C and H.

### Molecular Electrostatic Potential of the TMA Cation

**Figure 6:**
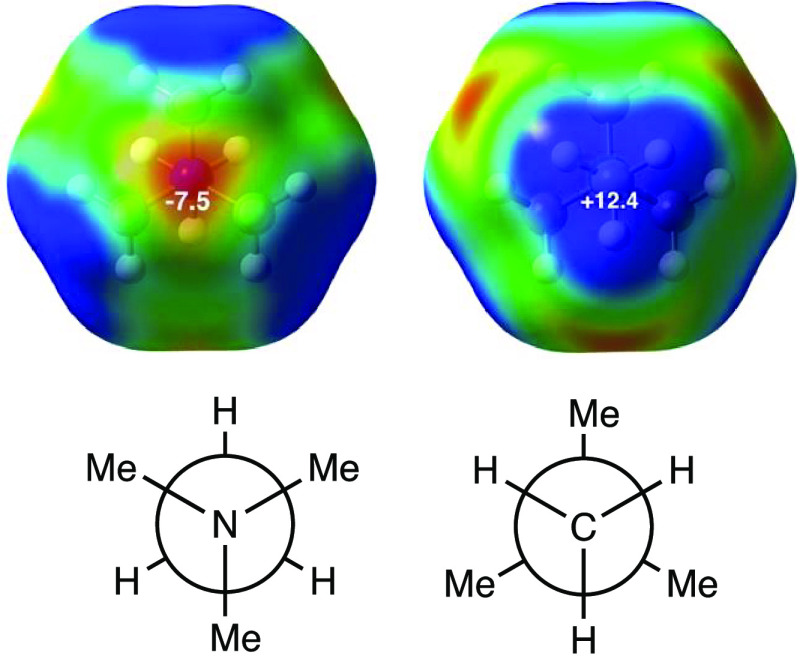
MEP maps of the TMA cation in two different orientations, calculated at the M062X/def2-TZVP level of theory, showing the most negative (red) and most positive (blue) regions of the electrostatic potential. The energy values at *V*_s,min_ and *V*_s,max_ are given in kcal/mol. (Right) The generalized-cuboctahedral shape of the molecule.

The molecular electrostatic potential (MEP) of the TMA cation (Figure 6) shows clearly its highest positive regions (*V*_s,max_ = +12.4 kcal/mol) at the center of the outer tetrahedral face of each of the methyl groups, which is consistent with the abundance of κC contacts of halides with methyl groups of alkylammonium cations since such an arrangement maximizes Coulombic attraction between the two ions. On the other hand, there is a region of negative electrostatic potential in TMA (*V*_s,min_ = −7.5 kcal/mol), associated with the more electronegative nitrogen atom, which may generate some degree of electrostatic repulsion in the 3κH contacts, although it should be expected to be rather weak due to the long X···N distances in those ion pairs (the shortest X···N distances found are 3.34, 3.62, 3.87, and 4.11 for F, Cl, Br, and I, respectively), which are found to be in all cases significantly longer than the sum of van der Waals radii: 2.86, 3.48, 3.49, and 3.70; i.e., penetrations of 14, 0, 2, and 0% for F, Cl, Br, and I, respectively.

### Computational Analysis

**Figure 7:**
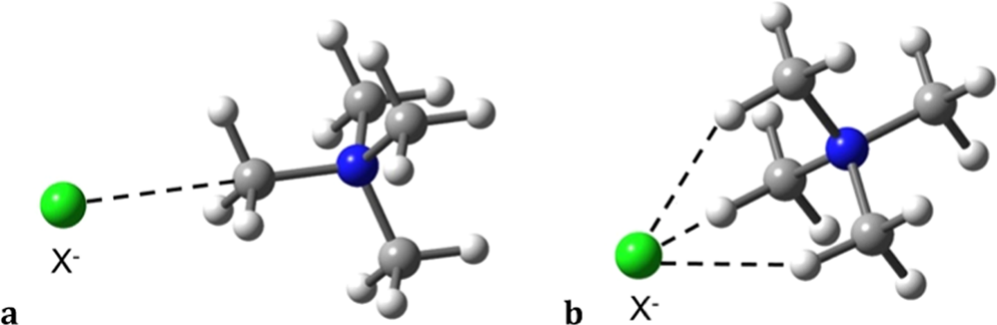
Models used to study κC (a) and 3κH **(**b) X^–^···[CH_3_N(CH_3_)_3_]^+^ ion pairs.

Our structural analysis indicated that two types of noncovalent interactions are mainly responsible for the crystal packing of (TMA)X compounds. Accordingly, we have created two simple models to comprehensively analyze the nature and strength of the κ^1^C and 3κ^1^H adducts (Figure 7). Five monoatomic anions have been used, namely, fluoride, chloride, bromide, iodide, and auride. In the next sections, different computational tools will be applied to understand the main characteristics of both interaction types. Optimization of the κ^1^H and κ^2^H ion pairs for X = Cl led to the 3κH and κC geometries, respectively, indicating that such interaction types may appear when the triple contacts are prevented due to packing requirements or by the nature of the ammonium ions, as in H_3_N–C(Me,Ph)H···Cl,^23^ in which there is only one hydrogen atom available.

### κC and 3κH Ion Pairs

The main results for the optimized models with these interaction topologies are summarized in Table 2. The binding energies for the ion pairs vary with the interacting topology and with the nature of the anions between −73 and −122 kcal/mol. Such high values are not surprising for an essentially strong Coulombic interaction, are similar to those previously determined for related systems,^24−26^ and differ by less than 3 kcal/mol from those reported for the two topologies of the chloride and bromide by Denisov et al.^27^ It is noteworthy that the binding energy of every 3κH ion pair is systematically higher (by 14–25 kcal/mol) than for the corresponding κC adduct.

**Table 2:** Calculated Data for the Optimized Structures of the κC–X···H_3_CNMe_3_ and 3κH–X···Me_3_NCMe (X = F, Cl, Br, I, Au) Ion Pairs Optimized at the M062X/def2-TZVP Level of Theory: BSSE-Corrected Binding Energies (kcal/mol), Contact Distances (Å) and Angles, van der Waals Penetrations *p*_AB_ (%), and Dipole Moments μ (D)^a^

	X	Δ*E*_BSSE_	X···C	*p*_XC_	X···H	*p*_XH_	*p*_XN_	C_int_–N	C_ni_–N	μ (D)
κC										
	F	–96.57	2.204	54	2.148	14		1.561	1.479	13.68
	Cl	–81.30	2.819	43	2.665	21		1.525	1.484	17.11
	Br	–77.45	2.998	38	2.824	15		1.520	1.485	18.03
	I	–73.30	3.225	35	3.032	14		1.517	1.486	19.10
	Au	–74.14	3.076	51	2.904	33		1.528	1.485	17.95
3κH										
	F	–122.09	2.756	25	1.822	47	12	1.498	1.478	9.20
	Cl	–99.48	3.322	15	2.347	40	–2	1.497	1.482	12.24
	Br	–93.61	3.509	7	2.523	35	–12	1.497	1.482	13.19
	I	–87.43	3.746	4	2.746	32	–17	1.496	1.483	14.29
	Au	–87.87	3.595	25	2.598	50	0	1.496	1.483	13.21

a *C*_int_ and *C*_ni_ refer to the carbon atoms bonded to H atoms interacting and not interacting with the anion, respectively.

To test the effect of the solvent on the calculated binding energies, we recalculated them for the case of (TMA)Cl within the continuous polarizable model (PCM) with three solvents of increasing polarities, chloroform, dimethylformamide, and water. As expected, the results (Table S2 in the Supporting Information) indicate a slight stabilization of the two independent ions and a corresponding increase of the binding energies of both d 3κ^1^H adducts by 2–5 kcal/mol. The relative stability of the two binding modes, however, is practically unaltered, and the κ^1^C ion pair is slightly more stable (by −19.25 kcal/mol) in the presence of water, the most polar solvent, than in the gas phase (−18.18 kcal/mol).

**Figure 8:**
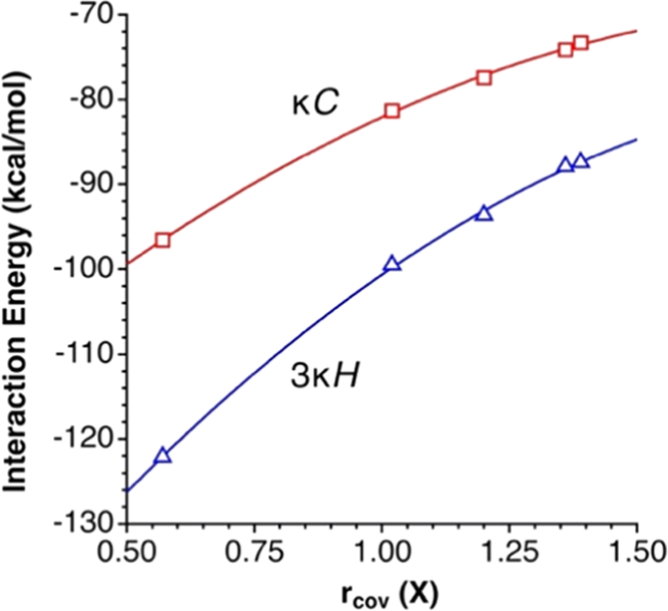
Calculated binding energy between an anion X^–^ and the TMA cation in two interaction topologies, represented as a function of the atomic covalent radius of the anion (X = F, Cl, Br, I, Au).

The pairing energies of TMA with the different anions (Table 2) are clearly higher for the fluoride and decrease as we go down the periodic group. Still more interesting, the auride adducts seem to follow the same trend, and a nice correlation of the binding energy and the atomic radius^28^ of the anion is thus found for both the κ^1^C (*E*_int_ = −123.8 + 55.98*r*_cov_ – 14.24*r*_cov_^2^) and 3κ^1^H ion pair families, (*E*_int_ = −161.4 + 80.01*r*_cov_ – 19.24*r*_cov_^2^), as seen in Figure 8.

The analysis of the optimized structures of the ion pairs shows that the optimized interatomic X···H contact distances are significantly shorter than the van der Waals radii sum in all cases, yet there are differences between the two topologies, with the κC pairs having distances about 0.3 Å shorter to a given anion than the 3κH ones. An obvious consequence of the short distances is that in all cases, there are positive *p*_XH_ penetration indices.

The optimized geometries for the two types of contacts present clear angular preferences, placing the anion in one of the trigonal axes of the TMA cation (**7** and **9**). Effectively, the X···C–N angle in the κC adducts and the X···N–C angle in the 3κH ones all are within the range of 178.6–180.0°. In the case of the κC ion pairs, thus, the anion X sits in the region of the maximum positive electrostatic potential (Figure 6), as happens in σ-hole interactions. In the case of the 3κH topology, the anion seems to be facing a region of negative electrostatic potential, disqualifying a simple electrostatic attraction, although this issue will be analyzed in more detail below.

Since the closest groups to the anion are different in the two geometries, we discuss now the interpenetration of the van der Waals spheres separately in the following.

**Figure 9:**
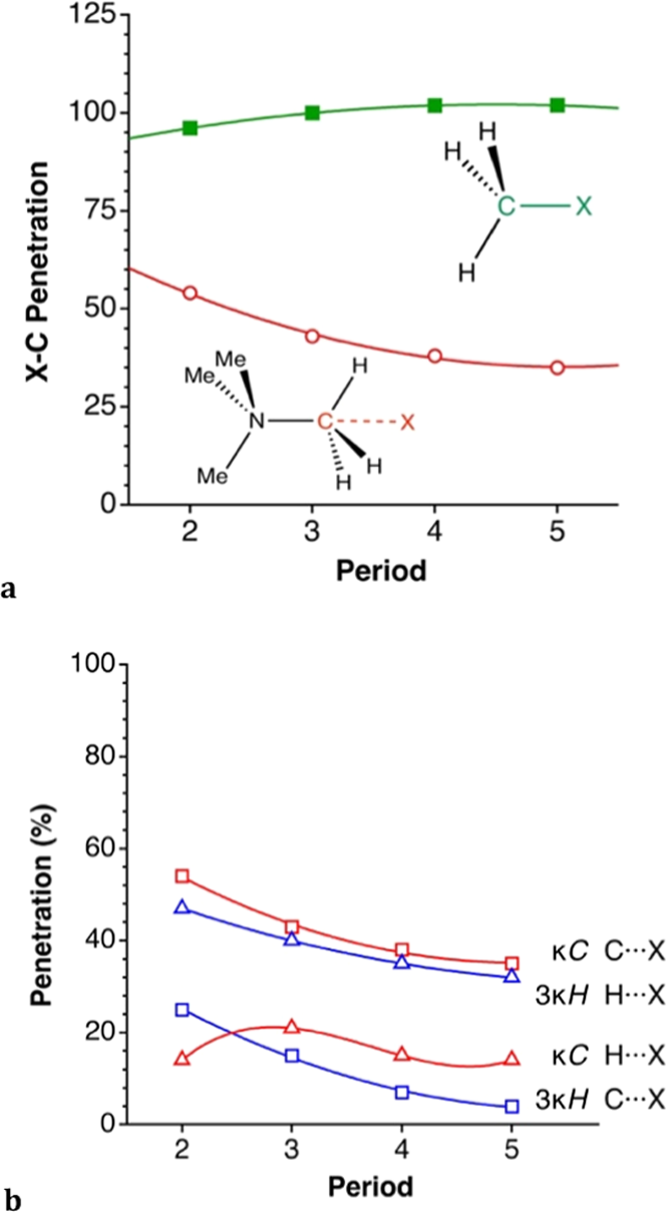
Penetration of the van der Waals crusts of the ion pairs represented as a function of the period for (a) the halogen and carbon atoms in the κC contacts (open circles) and the corresponding values for the X–C bonds in methyl halides (solid squares). (b) Comparison of the penetration of the hydrogen (triangles) and carbon (squares) atoms of tetramethylammonium with the anions for the two interaction topologies.

In the κC ion pairs, the penetration indices for the X···H contacts appear in the range 14 ≤ *p*_XH_ ≤ 21 for the halides, whereas a significantly larger value is found for the auride anion. Note that, even if the distance of a given anion to the C atom of the interacting methyl group is about 0.1–0.2 Å longer than the X···H distances, the penetration of the carbon van der Waals crust is significantly larger for the carbon atom (Table 2), with penetration indices in the range 35 ≤ *p*_XC_ ≤ 54%. This geometrical feature indicates that the carbon atom plays a major role in the interaction with the anion and justifies the use of the κC notation. If we focus on the halides, nice nonlinear inverse correlations are found for the penetration index *p*_X_ as a function of either the X atomic number or the period in which it appears in the periodic table. The latter can be appreciated in Figure 9a, where it can be compared with the penetration in the related covalent X–C bonds of methyl halides.^29,30^ Three remarkable aspects of that plot must be highlighted. First, all of the halides (and auride as well) clearly penetrate the van der Waals crust of the methylic carbon, but far from the full penetration (100%) required to form a bond. Second, the degree of penetration decreases as we go down the group of halogens. Third, a comparison with the related X–C bonds in methyl halides clearly illustrates the difference between a bond and a noncovalent interaction. Surprisingly, the dependence of the penetration index on the position of the halogen atom in the group is just the opposite for the bonds than for the ion pairs. The penetration of the H’s crust by the halides is significantly smaller but non-negligible in most cases (Figure 9b).

**Figure 10:**
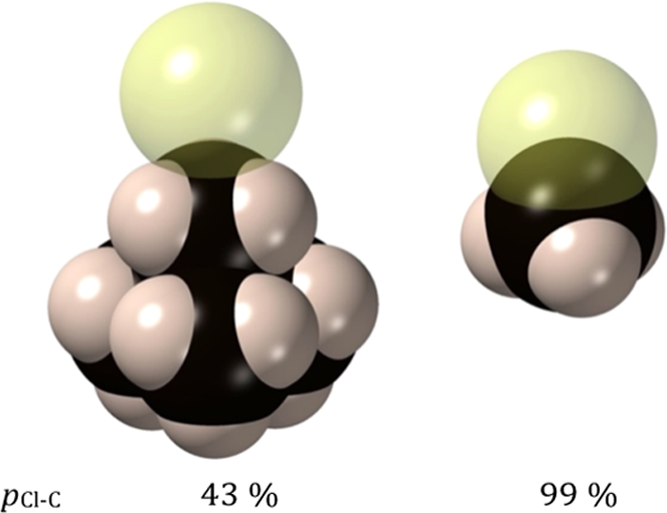
Representation of the atomic van der Waals spheres of the optimized structure of the κC–Cl···H_3_CNMe_3_ ion pair (left), showing the larger penetration of the fluoride into the van der Waals crust of the carbon atom compared to hydrogen atoms. (b) Similar representation of the MeCl molecule^31^ for comparison of the different degrees of penetration of a C···Cl contact and a C–Cl covalent bond.

The different X–C penetrations of the van der Waals crusts in the halomethanes compared with the TMA halides can be appreciated as well in the molecular models of Cl–CH_3_ and Cl···H_3_CNMe_3_ (Figure 10). Although the ratio of the two penetration indices is somewhat larger than 2, the visual impression is that the overlapping volume in the covalent bond is much more than twice that observed in the ion pair. Certainly, the penetration index is a one-dimensional variable, while the volume is expected to have a cubic dependence on the radius of the overlapping region, assuming that the intersection had a spherical form. As a matter of fact, the intersection has the shape of a lentil, and this issue will be dealt with in more detail in a forthcoming publication, although we can advance that such a volume can be expressed as a nonlinear function of the penetration index. Thus, the penetration index is an easy-to-calculate parameter that can be applied in principle to any pair of atoms and allows for direct comparison of two different combinations of elements, while at the same time it bears some relationship to the amount of volume shared by the two van der Waals crusts. It is worth pointing also to the small overlap of the chlorine and hydrogen van der Waals spheres in κC–Cl···H_3_CNMe_3_ compared to that between the chloride and carbon atoms (Figure 10, left).

In the alternative 3κH arrangement, the penetration of the hydrogen by a given anion is roughly between 2 and 3 times higher than in the κC interactions. On the contrary, the penetration of the carbon atoms is from 2 to 10 times smaller, as can be seen in Figure 9b. At this point, it is reasonable to ask whether there is some interpenetration of the halide and nitrogen crusts in the 3κH case, in which there are no intervening atoms between them. The *p*_XN_ values are in most cases either zero (X = Au) or negative (−2, −12 and −17% for X = Cl, Br and I, respectively), indicative of no interpenetration, except for X = F, for which there is a penetration of 12% despite its smaller size.

The calculations including the solvent effect via the PCM model for the case of (TMA)Cl result in larger cation–anion separations, reflected in smaller Cl···C and Cl···H penetration indices, in better agreement with the values found from X-ray crystallography data (Figure 5). The highest penetration index in the κC chloride, *p*_C–Cl_, drops from 43% in the gas phase to 18% in the presence of water, while in the 3kH chloride, it is the *p*_H–Cl_ index that falls from 40 to 21% (see Figure S3 in the Supporting Information).

**Figure 11:**
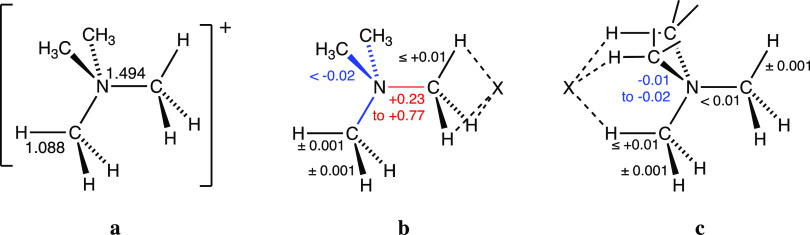
Bond distances in the independent TMA cation with tetrahedral symmetry (a), and changes experienced by them upon ion pair formation with an anion X in the κC (b) and 3κH (c) interaction modes. All values in Å.

The σ-hole interactions are characterized by the existence of electron density donation from a Lewis base to an empty σ* orbital corresponding to a bond trans to the noncovalent interaction, in the present case, the C–N bond. As a consequence, one should expect some weakening of that bond. The relevant changes in bond distances upon ion pair formation are summarized in Figure 11. Interestingly, in the five κC ion pairs investigated, the C–N bond corresponding to the interacting methyl group is significantly lengthened (0.23–0.77 Å), whereas the noninteracting C–N bonds present a minute contraction (by 0.02 Å or less). In the alternative 3κH topology, the changes in all bond distances are minimal (0.02 Å or less) for both the interacting and noninteracting methyl groups. Variations in the N–C–H bond angles upon ion pair formation are within chemical accuracy except for κC F···H_3_CNMe_3_, for which the three contacting hydrogens are bent away from the fluoride by 2°, and 3κH F···Me_3_NCH_3_, in which the interacting H atoms form smaller N–C–H bond angles, 4° smaller than the tetrahedral angle, to get closer to the fluoride.

To simplify the discussion, the upper and lower limits of the changes in bond distances of the TMA cation upon ion pair formation are summarized in Figure 11, whereas the bond distances of the TMA cation, free and in the ion pairs, are provided in the Supporting Information (Table S1 and Figure S1). The first thing that must be said is that the calculated X···C distances in these ion pairs are significantly shorter—and the penetration indices accordingly larger—than in the experimental structures, a trend that can be attributed to the fact that such a cation in the solid state does not form ion pairs but establishes similar interactions with a larger number of counterions (see, e.g., Figure 2) in three-dimensional networks, an issue that will deserve more attention in a moment.

In Figure 11, we can see that the most dramatic structural change observed corresponds to an elongation of the C–N bond by 0.23–0.77 Å in the κ^1^C ion pairs (Figure 11b), while other bonds are very little affected, as expected for an electron density donation from the anion to a σ*(N–C) molecular orbital, characteristic of σ-hole interactions. In the alternative 3κH mode (Figure 11c), in contrast, all changes in the bond distances are of at most 0.02 Å.

### Changes in the Atomic Charge Distribution

**Figure 12:**
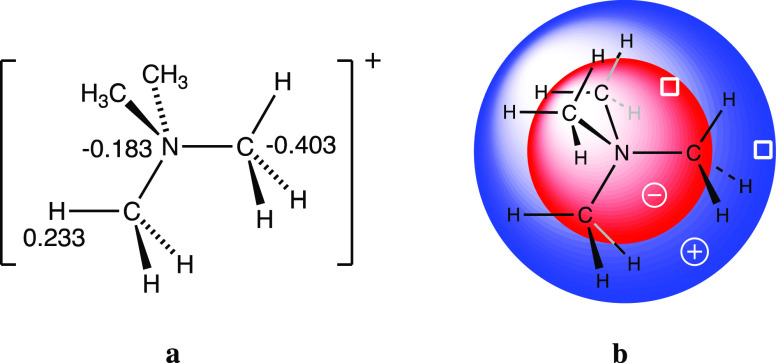
Calculated atomic charges in the independent TMA cation (a) and schematic depiction of the pseudospherical charge distribution (b). Note that all carbon atoms are equivalent by symmetry within chemical accuracy, and likewise for the hydrogen atoms. The white squares indicate the approximate positions of the maximum positive and negative electrostatic potentials seen in Figure 6.

Given the expected dominant Coulombic character of the interaction between the two ions, it is worth taking a look at the charge distributions in the ion pairs, obtained from a natural population analysis (NPA), even if the numerical values should be taken with a grain of salt. The net atomic charges for the independent TMA cation (Figure 12a) show that the inner NC_4_ tetrahedron holds a negative charge of −1.80, concentrated mostly at the carbon atoms and, therefore, a direct X···C interaction is electrostatically disfavored, an issue that will be dealt with in more detail below. All of the positive charge of (+2.80) is distributed among the outer shell of 12 hydrogen atoms, providing us with a raw description of the TMA cation as formed by a sphere of negative charge circumscribed in a spherical shell of positive charge (Figure 12b).

**Figure 13:**
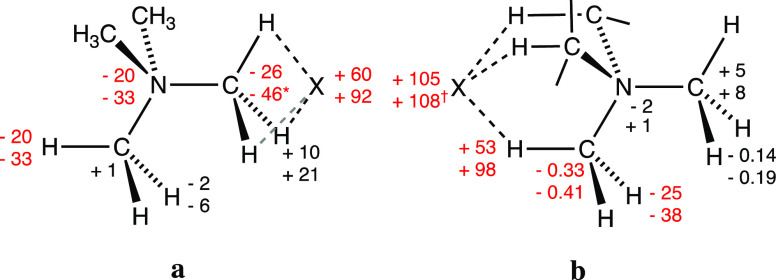
Changes in atomic charges (in me^–^) upon pair formation for the five groups of symmetry-related atoms in the C_3v_ point group for the κC (a) and 3κH (b) interaction modes. The most significative values are given in red, and two values that fall well outside the ranges shown are (*) the charge of the κC atom in the fluoride that loses 29 me of its negative charge and (†) that of the auride anion in the 3κC adduct, that releases a larger amount of electron density (135 me) than the halides.

Upon ion pair formation, the charges of the different atoms (see Figure S2 in the Supporting Information) are modified within the ranges shown in Figure 13a,b for the κC and 3κH models, respectively. In the κC pairs, it must be remarked that the interacting carbon atom becomes still more negatively charged, even if they are those with the highest negative charge in TMA. Negative charge transfer in that topology extends as well to the N atom and to the distal H atoms. It is also remarkable that the contacting hydrogen atoms increase their positive charge.

Note that the MEP map (Figure 6) presents the electrostatic potential at a given isosurface of electron density and is therefore showing points at different distances from the N atom along the two different trigonal axes, indicated by squares in Figure 12b. This explains why the 3κH contact is electrostatically attractive despite the negative potential shown by the MEP map (Figure 6) since the anion interacts with the outer region of positive electrostatic potential rather than with the inner negative shell, as indicated by the high *p*_XH_ penetration indices compared to *p*_XC_ and *p*_XN_ (Table 3).

**Table 3:** Energy Decomposition Analysis of the TMA···X Interactions for the κC- and 3κH-Ion Pairs, and NBO Stabilization Energies *E*^(2)^ for the LP (X) → σ*(C–N) and LP (X) → σ*(C–H) Donor–Acceptor Interactions in the κC and 3κH Systems, Respectively^a^

	anion	Δ*E*_int_	Δ*E*_Pauli_	Δ*E*_elec_	Δ*E*_disp_	Δ*E*_pol_	Δ*E*_CT_	*E*^(2)^
κC								
	F^–^	–96.57	32.40	–104.45	–5.76	–12.61	–6.14	18.08
	Cl^–^	–81.30	19.42	–85.17	–4.99	–6.25	–4.31	10.84
	Br^–^	–77.45	22.64	–85.50	–4.83	–5.74	–4.00	9.45
	I^–^	–73.30	19.07	–77.69	–5.60	–5.08	–4.00	7.87
	Au^–^	–74.15	26.02	–83.33	–5.99	–5.81	–5.03	8.33
3κH								
	F^–^	–122.09	46.38	–133.59	–8.39	–20.42	–6.07	18.2
	Cl^–^	–99.49	31.06	–104.74	–7.44	–10.86	–7.49	18.4
	Br^–^	–93.61	29.34	–98.69	–7.09	–10.09	–7.07	17.6
	I^–^	–87.43	28.93	–91.97	–8.30	–8.82	–7.28	16.2
	Au^–^	–87.89	39.95	–99.80	–8.89	–9.81	–9.34	21.5

a All values in kcal/mol, calculated at the M062X/def2-TZVP level of theory.

**Figure 14:**
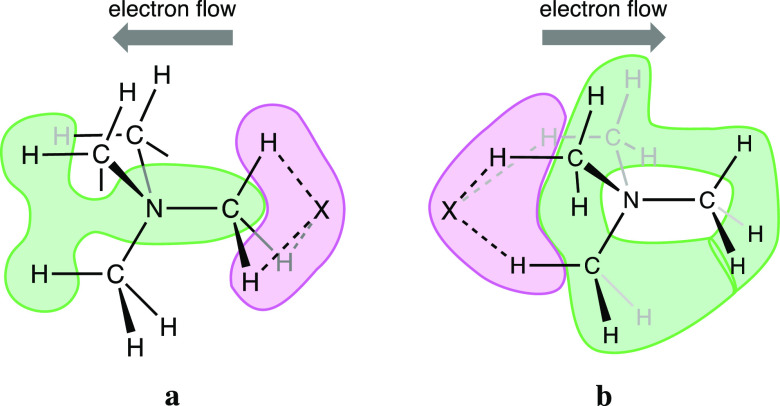
Regions of depletion (green) and accumulation (pink) of electron density upon ion pair formation in the κC (a) and 3κH (b) interaction modes.

Comparison of the charge distribution in the κC and 3κH interactions can be represented by a cartoon (Figure 14) that reveals the different paths that follow the electrons donated from the halide and the contacting hydrogen atoms to the cation in the two cases. In the κC interactions (Figures 13a and 14a), the electron donation goes to the C–N bond that defines the trigonal axis and the distal hydrogen atoms, whereas in the 3κH case (Figures 13b and 14b), the C–N axis remains practically unaltered, and the electron population increases in both the distal and proximal hydrogen atoms. The fact that the amount of negative charge shift from the anion to the TMA cation is in all cases twice in the 3κH interactions than in the κC ones (Figure 13) agrees well with the stronger dipole moment (μ, Table 1) in the former case, to be compared with the almost negligible value calculated for the independent TMA cation. We must recall, however, that the present results are not representative of what happens in the solid-state TMA salts, in which the cation establishes interactions with several anions in the three directions of space.

### Energy Decomposition and Natural Bond Orbital Analyses

An energy decomposition scheme^12^ provides information on the relative contribution of different terms to the total interaction energy. Those terms are (a) Δ*E*_Pauli_, corresponding to the repulsion between paired electrons of the two interacting fragments, which includes as well the core–core repulsions; (b) an electrostatic term Δ*E*_elec_; (c) a charge transfer term Δ*E*_CT_ corresponding to donor–acceptor interactions; (d) Δ*E*_pol_, a polarization term resulting from mixing of occupied and empty orbitals of one fragment induced by the presence of the other fragment; and (e) a dispersion term Δ*E*_disp_, reflecting the London forces acting between the two fragments. Since the charge transfer and polarization terms involve both modifications of the molecular orbitals upon interaction, we will refer to the sum of the two contributions as an overall orbital contribution Δ*E*_orb_, while the terms related to the Pauli and electrostatic interactions will be summed up in a single Pauli + electrostatic term, Δ*E*_Pe_. The results of the energy decomposition analysis (EDA) are presented in Table 3 for the five anions under consideration in the two topologies found to correspond to minima in the potential energy surface.

Consistently with the electrostatic interaction expected from the charge distribution in the case of the κC ion pairs, we can see that for every anion, the stabilizing contribution of the electrostatic attractive term is more than 10 kcal/mol higher for the alternative 3κH topology.

**Figure 15:**
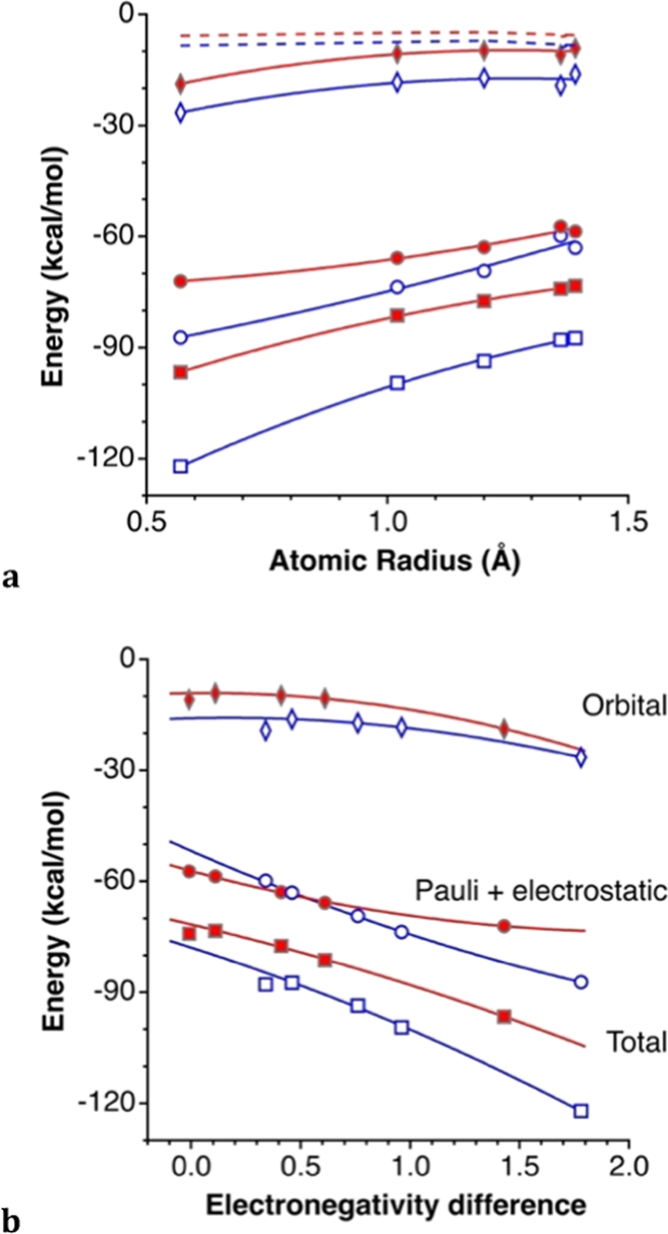
(a) Interaction energies (squares), dispersion (dashed lines), orbital (diamonds), and Pauli + electrostatic (circles) contributions for the κC- (filled symbols) and 3κH- (empty symbols) topologies in the TMA···X ion pairs (X = F, Cl, Br, I, and Au) as a function of the atomic radius. (b) Dependence of the energies on the Pauling electronegativity differences between X and the C or H atom for the κC (squares) and 3κH (triangles) ion pairs, respectively.

By looking at the interaction energies and their EDA results (Figure 15), several clear conclusions can be drawn:

(1) For a given anion, the 3κH interacting mode is significantly more stabilizing than the κC one.
(2) For the same interaction topology, the stability of the ion pair increases as the monoatomic anion becomes smaller (or, equivalently, more electronegative).
(3) The most important contribution in both interaction modes comes from the sum of the Pauli and electrostatic terms, as expected for an ionic interaction.
(4) The orbital contribution is much smaller but non-negligible, and significantly higher in the 3κH interacting mode.
(5) The dispersion contribution is rather small and practically independent of the nature of X.
(6) The orbital and Pauling + electrostatic contributions show a similar dependence on the nature of X than the total interaction energy.

**Figure 16:**
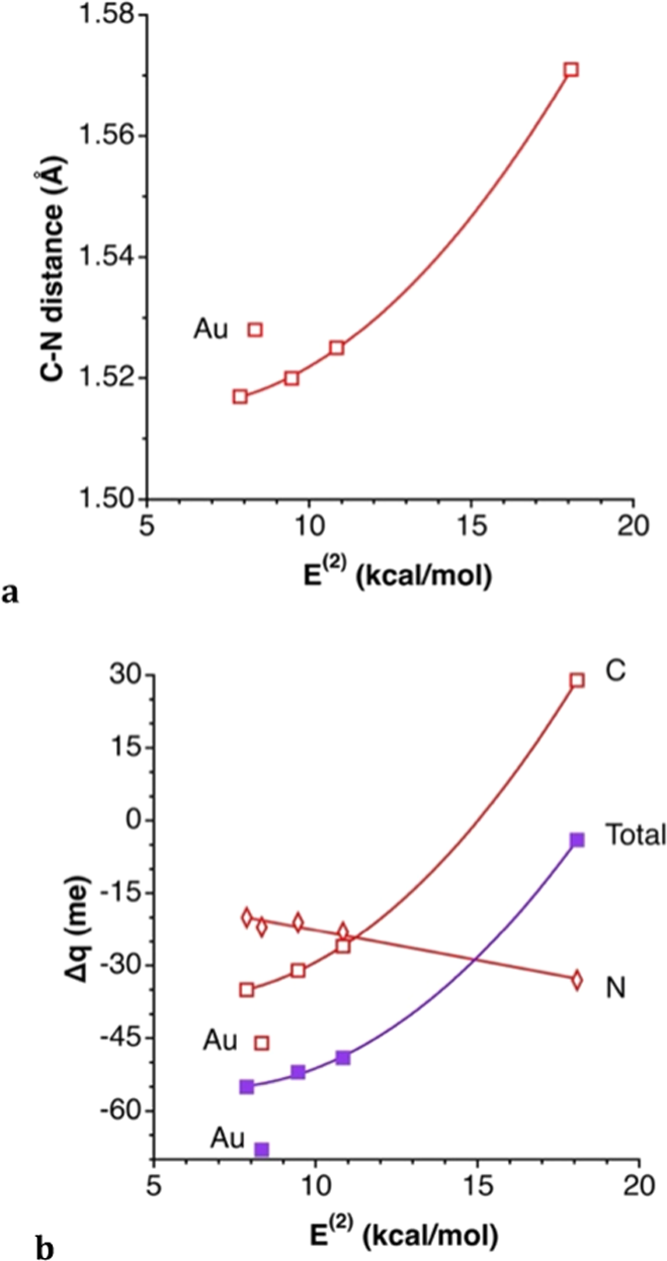
Relationships of (a) the proximal C–N bond distance and (b) the charge variations of those C and N atoms upon adduct κC ion pair formation with the stabilization energy *E*^(2)^ associated with the LP(X) → σ*(C–N) donor–acceptor interaction in the κC ion pairs. The lines shown are least-squares fittings disregarding the auride.pon adduct formation. The lines shown are least-squares fittings disregarding the auride.

**Figure 17:**
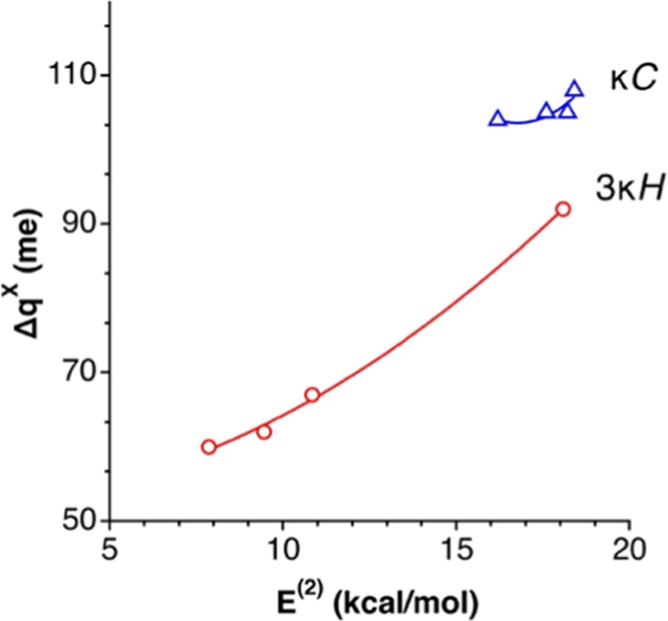
Relationships between the variation of the net charge of the halide X, Δ*q*^X^, and the energy *E*^(2)^ associated with the LP(X) → σ*(C–H) donor–acceptor interactions in κC (circles) and 3κH (triangles) ion pairs.

A natural bond orbital (NBO) analysis of the κC ion pairs discloses the existence of one donor–acceptor orbital interaction from an X lone pair to a C–N antibonding orbital, LP(X) → σ*(C–N), with an associated stabilization energy *E*^(2)^ from 7.87 to 18.08 kcal/mol (Table 3), with a significant decrease as the atomic number of the anion increases, a trend that can be correlated with nonlinear dependence on the Pauling electronegativity or on the covalent radii of the anion. Moreover, those stabilization energies present an excellent linear correlation with the total interaction energy (*E*_int_ = −55.3–2.274 *E*^(2)^, *r*^2^ = 0.996). Such an electron density donation is reflected in a lengthening of the C–N bond as the interaction becomes stronger, showing a nice correlation for the TMA halides (Figure 16a). Since the σ*(C–N) molecular orbital has a much larger contribution at the carbon atom, due to its lower electronegativity, this donor–acceptor interaction also increases the negative charge of the interacting carbon atom (Figure 16b), while a small decrease of the negative charge at N is most likely due to a second-order effect, i.e., a rehybridization of the σ(C–N) and σ*(C–N) MOs. Consistently, the negative charge of the halide anions also decreases as the donor–acceptor interaction strength increases (Figure 17).

The corresponding second-order perturbative analysis for the 3κH interaction topology reveals the existence of donor–acceptor interactions between the halide anions and the σ*(C–H) MOs. The calculated stabilization energies *E*^(2)^ (Table 3) indicate that there is significant electron delocalization from the halide or auride anion to the cation in all cases, in agreement with a higher loss of negative charge of the anions, compared to the κC analogues (Figure 17) and the significant increase of the negative charge at the carbon atoms (Figure 13). The set of three X···H contacts results in a higher stabilization compared with the κC ion pairs, and the strength of the interaction is very little affected as we move down the halogen group (Table 3).

**Figure 18:**
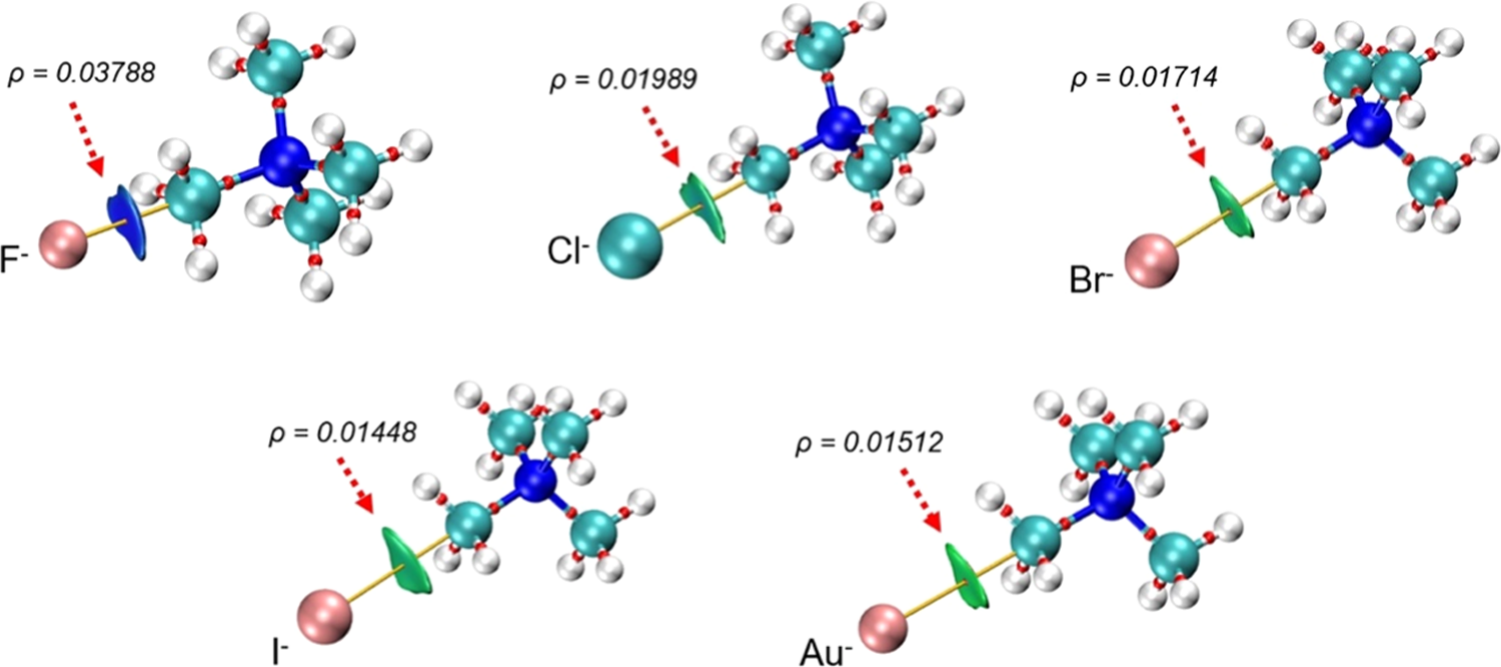
Combined QTAIM (bond critical points and bond paths) and NCI isosurfaces for κC–X···TMA ion pairs (X = F, Cl, Br, I, Au) at the M062X/def2-TZVP level of theory.

For a complementary view of the bonding interactions in the κC–X···H_3_CNMe_3_ (X = F, Cl, Br, I, Au) ion pairs, a Bader’s quantum theory of atoms in molecules (QTAIM) analysis was performed. The topological parameters such as electron density (ρ), Laplacian of electron density (∇^2^ρ), total energy density (*H*), and kinetic energy density over electron density (*G*/ρ) were computed and are provided in the Supporting Information (Table S3). The QTAIM results evidence that both the halides and auride anions are indeed connected to the carbon atom of the methyl group by a bond path, consistent with the presence of an X···C σ-hole interaction (Figure 18). The electron densities at the bond critical points (bcp) follow the order I ≈ Au < Br < Cl ≪ F (Figure 18). The F···C contact exhibits by far the highest value of bcp electron density, thus indicating a significant covalent character of this contact. This interpretation agrees with the trends of the covalent components found in the EDA above, and the two parameters present a perfect correlation for the four halides. In addition, in the NCI (noncovalent interaction) isosurface plot (Figure 18), the blue color of the isosurface between C and F is consistent with the more covalent nature of the F···C contact. The σ-hole X···C contacts for the other halogens and for auride are further characterized by small and green NCI plot isosurfaces located between the two interacting atoms and coincident with the bcp. On the other hand, the Laplacian of the electron density and the kinetic energy density over electron density are positive, which is an indication of the existence of strong electrostatic interactions. In accordance with the classification proposed by Grabowski et al.,^32^ the noncovalent interactions show positive values of ∇^2^ρ and the total energy density H.

**Figure 19:**
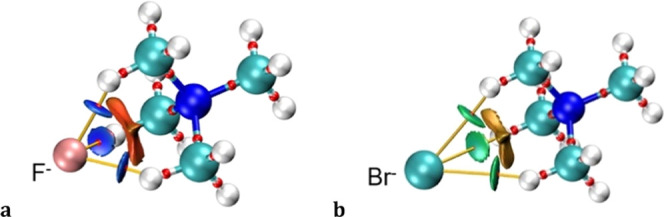
Combined QTAIM (bond critical points and bond paths) and NCI isosurfaces of 3κH (a) F···TMA and (b) Br···TMA_3_ complexes.

A similar QTAIM analysis was carried out for the 3κH–X···Me_3_NMe (X = F, Cl, Br, I, Au) ion pairs. The H···X interactions appear in the topological analysis of the electron densities as bcp’s in bond paths connecting the anion and the H atoms (Figure 19). Additionally, NCI plots (Figure 18) present blue (X = F) and green (X = Cl, Br, I, Au) isosurfaces that confirm the attractive nature of these interactions. Interestingly, these regions of attractive interaction coincide with an electrostatically repulsive X···N region seen in the MEP plot of the TMA cation (Figure 6). However, since the interpenetrations of the X and N van der Waals crusts are rather small or negative (−17 ≤ *p*_XN_ ≤ 12%), compared to those of the three electrostatically attractive X···H interactions (32 ≤ *p*_XH_ ≤ 50%), the net electrostatic contribution to the interaction energy is much more attractive than in the κ^1^C case, according to the EDA (Table 3). It is interesting to note that, even if the electrostatic contribution is stronger for the 3κH than for the κC ion pairs, so are the covalent ones, resulting in an average 20 (2) % of the attractive forces coming from the orbital terms, in agreement with the donor–acceptor interactions found in the NBO analysis, and to be compared to a slightly smaller proportion in the κC case, 14 (2) %.

**Figure 20:**
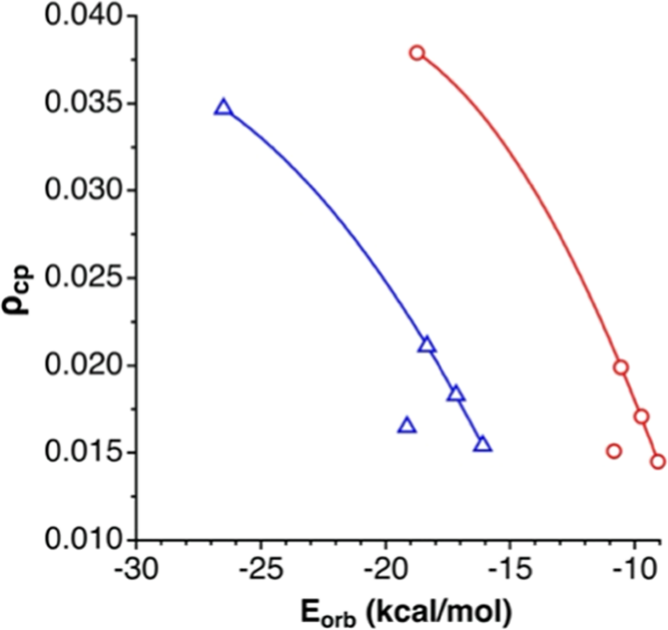
Relationship between the electron density at the X···C and X···H bond critical points for the κC and 3κH ion pairs, respectively, and the orbital contribution to the interaction energy found in an EDA. The outliers correspond to the aurides, and the curves shown are least-squares fittings of the data for halides only.

As can be seen in Table 6, the positive values of the Laplacian of the electron density observed for the H···X interactions are characteristic of closed-shell interactions. The topological parameters reported in Table S3 indicate that the strengths of the H···X bonding follow the order H···F > H···Cl > H···Br > H···Au > H···I and the electron densities at the bond CP are linearly correlated with the H···X intermolecular distances. Similarly to that found for the κC adducts, the electron density at the BCP increases in the order I ≤ Au < Br < Cl ≪ F (0.0154, 0.0165, 0.0183, 0.0211, and 0.0347, respectively), which for the halides follows the same order as the penetration indices. These densities are as well correlated with the orbital contribution found in the EDA (Figure 20) for the halides, while the auride somewhat deviates from the general trend.

## Conclusions

In the present computational and structural study of intermolecular interactions between the TMA cation and monoatomic halide and auride anions, we have introduced the concept of interpenetration of the van der Waals crust of two nonbonded atoms, and a parameter that calibrates the degree of interpenetration, the penetration index *p*_AB_. The symmetric interaction with an anion through only one methyl group is seen to present higher penetration indices with the carbon than with the hydrogen atoms, even if the X···H distances are shorter than the X···C ones. For that reason, such an interacting mode is labeled κ^1^C or, simply, κC.

The MEP map of the TMA cation presents a region of high positive electrostatic potential roughly at the center of the three hydrogen atoms of one methyl group, which is adequate for an attractive electrostatic attraction of an anion in the κC position. However, in the center of the triangle formed by the hydrogen atoms involved in the 3κH interaction, the electrostatic potential is negative and would predict unstable ion pairs based on purely electrostatic arguments. In contrast, both the κC and 3κH binding modes are found to correspond to minima in the potential energy surface of the optimized ion pairs. The binding energies vary from −73 to −97 kcal/mol for the κC and between −87 and −122 kcal/mol for the 3κH adducts, the latter topology being 14–25 kcal/mol more stable for the same anion. For each interaction type, the pairing energies show an excellent correlation with the covalent radii of the anions, becoming more negative as the atomic size decreases, thus making the fluoride ion pair the most stable within each series.

The changes in the charge distribution within the TMA cation upon ion pair formation are counterintuitive and completely different for the two interaction topologies studied. While the independent cation has a nearly spherical distribution of positive charge on the external hydrogen atoms and a negatively charged core formed by the NC_4_ group, the interaction with an anion enhances the positive charge of the nearby hydrogen atoms, while the approximately 0.1 electrons transferred to the cation accumulate mostly at the inner heavier atoms, following a distinct pattern in each of the interacting topologies.

The application of different theoretical approaches, EDA, NBO, QTAIM, and NCI plots, provides a consistent view of the nature of the TMA-X interaction. Thus, the most important contribution in both interaction modes comes from the electrostatic term in the EDA, as could be expected for an ionic interaction, which prevails over the Pauli repulsion term. The covalent contributions are much smaller but non-negligible. Both sets of components are more stabilizing in the 3κH interacting mode, making this topology more stable overall. The stabilizing dispersion contribution is comparatively small in all cases (between −5 and −9 kcal/mol) and practically independent of the nature of X.

The NBO analysis reveals the existence of relevant donor–acceptor interactions from the anion to a σ*(C–N) orbital in the κC case, and to a σ*(H–C) orbital in the 3κH geometry. Changes in the charge distributions and bond distances within the cation upon ion pair formation are consistent with the existence of such orbital interactions. Complementary, a QTAIM analysis discloses bond paths and bond critical points between X and the closest C atom in the κC ion pairs, and between X and the three closest H atoms in the 3κH adducts, while NCI isosurfaces are located between the two interacting atoms and coincident with the bcp’s. On the other hand, the Laplacian of the electron density and the kinetic energy density over electron density are positive which is an indication of the existence of strong electrostatic interactions. The electron density at the BCP increases in the order I < Br < Cl ≪ F, and are as well correlated with the covalent contribution found in the EDA (Figure 19) for the halides, while the auride somewhat deviates from the general trend, most likely due to the different role of its empty 6*p* orbitals compared to that of the occupied n*p* orbitals (*n* = 2–5) of the halides. Indeed, the NBO results indicate that the lone pair orbitals of the halides involved in the donor–acceptor interactions with TMA have a p orbital contribution of 87–92%, whereas in auride, the interacting lone pair has 88% d character.

Overall, we can see that the polarization and electrostatic energetic contributions, the net interaction energy, and the electron density at the intermolecular bond critical points decrease down the halogen group in the two types of ion pairs, the same trend is followed by the electronegativity difference between C and X (for the κC adducts), or between H and X (for the 3κH ones), and by the penetration indices *p*_XC_ and *p*_XH_, respectively. However, the charge transfer contributions that imply the interaction between a filled MO of the anion and an empty one of the cation show no clear dependence on the position of the latter within the group of the halogens, contrary to the orbital interaction rules.^33^ In fact, the strength of an orbital interaction is favored by a small energy difference between the interacting orbitals (roughly, the atomic electronegativities) and by a strong overlap. We may assume that the overlap between the valence orbitals of the interacting atoms can be roughly represented by the penetration index since it takes into account both the interatomic distance and the size of the atoms, essentially determined by the size of its valence orbitals. Then, the charge transfer interactions are favored for the heavier halogens with electronegativities closer to those of C and H, whereas they are disfavored due to the smaller penetration and the corresponding small overlap. Both factors seem thus to compensate, resulting in the very small variation of the Δ*E*_CT_ terms observed. Indeed, we can obtain a good bilinear correlation for the five 3κH ion pairs (eq 2, χ represents the electronegativity).
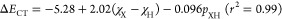
2
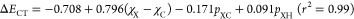
3

Although such a correlation must not be taken as statistically significant, due to the small number of data sets, it clearly shows the opposing contributions of the electronegativity difference and the penetration index. A similar analysis for the κC family, considering the most relevant penetration index in this case, *p*_XC_, gives poorer results. We recall, however, that in this topology, the X···H penetration, although smaller, is not negligible, and interaction with the H atoms may somewhat decrease electron donation to the C one. If we include both *p*_XC_ and *p*_XH_ in the regression, a reasonable expression results (eq 3), again to be taken with caution because of the small data set compared to the number of fitting parameters. In brief, while the Coulombic interaction dominates the anion–cation binding in the TMA-halide ion pairs, a non-negligible covalent contribution exists that can be associated with donor–acceptor interactions to N–C or C–H antibonding orbitals in the κC and 3κH adducts, respectively. The electrostatic interaction is clearly dependent on the X···H contacts because of both their opposed charges and shorter distances, a fact that accounts for the stronger Coulombic contribution in the 3κH ion pairs, as well as for its decreasing value as we descend down the halogens group. The covalent contribution, in contrast, depends on the electronegativity difference between the contacting atoms and the penetration index between them, resulting in a predominant participation of the carbon atom with higher penetration indices than the hydrogen ones in the κC topology. The opposite effects of the variations in the electronegativity and penetration indices down the group result in rather small changes in the covalent contributions to the interaction energies for the different anions explored.

### Computational Details Information

Structural searches were carried out in the Cambridge Structural Database (CSD), version 5.43 (November 2021).^22^ Crystal structures with 3D coordinates defined and nondisordered were considered. For the calculations of penetration indices, standard sets of covalent^28^ and van der Waals^34^ radii were used.

DFT calculations were carried out with the M062X functional and the def2-TZVP basis set^35^ for all atoms. This functional has shown very good performance dealing with noncovalent interactions in previous benchmark reports.^36,37^ Interaction energies were calculated via the supermolecule approach and corrected for the BSSE by means of the counterpoise method.^38^ MEP maps were built on the 0.001 Å electron density isosurface with GaussView.^39^ The polarizable continuum model (PCM) implemented in Gaussian16 was used to perform the calculations in the presence of different solvents. The dielectric constants employed for water, N,N-dimethylformamide, and chloroform were 78.355, 37.219, and 4.771, respectively. QTAIM and NCI topological analyses of the electron density were performed using the Multiwfn 3.7 program^40^ on the corresponding DFT wave functions and represented using VMD.^41^ NBO analysis was done with the NBO 3.1 software^42^ as implemented in Gaussian16^43^ at the DFT level. For the decomposition of the interaction energy, we employed the second-generation ALMO-EDA method^44^ implemented in Q-Chem 5.3.^45^ All other DFT calculations were done with Gaussian16.^43^

## Supporting Information Available

Selected bond distances in TMA and its changes in the ion pairs, QTAIM topological parameters of the electron densities, atomic coordinates of the optimized structures, and calculated atomic charges (PDF)
