# Supplementary material for: Tetramethylammonium Cation: Directionality and Covalency in Its Interactions with Halide Ions
Source: Inorg Chem. 2022 Jun 6;61(24):9082–95. doi: 10.1021/acs.inorgchem.2c00600 (PMC9214700; doi:10.1021/acs.inorgchem.2c00600)
Supplement: Supplementary file 1 — ic2c00600_si_001.pdf [file ic2c00600_si_001.pdf]

The Tetramethylammonium Cation: Directionality and Covalency in its Interactions with Halide Ions

Diego M. Gil, Jorge Echeverría and Santiago Alvarez

Supporting Information

	page
**Table S1**. Bond distances within the TMA cation in κ^1*C*-TMA···X ion pairs	S2
**Table S2**. Binding energy for the κ^1*C* and 3κ^1*H*-(TMA)Cl ion pairs in different solvents	S2
**Table S3.** QTAIM topological parameters at bond CPs for κ*C* X··· TMA ion pairs	S3
**Table S4.** QTAIM topological parameters at bond CPs for 3κ*H* X···TMA ion pairs	S3
**Table S5**. Atomic coordinates of the optimized structures of the κ*C*-X···TMA ion pairs	S4
**Table S6**. Atomic coordinates of the optimized structures of the 3κ*H*-X···TMA ion pairs	S6
**Figure S1**. Optimized bond distances in the TMA cation and its ion pairs	S9
**Figure S2**. NPA atomic charges calculated for the X···TMA ion pairs	S10

Table S1. Bond distances (Å) within the TMA cation, isolated and in κ[1]*C*-TMA···X ion pairs, and changes in those distances in the interacting and non-interacting N-CH_3 groups upon coordination. The C-H distances of the non-interacting methyl groups (not given) change by, at most, ± 0.001 Å.

X	C-N_{int}	δ(C-N_{int})	C-N_{non}	δ(C-N_{non})	C-H_{int}	δ(C-H_{int})
none			1.494		1.088	
F	1.571	0.077	1.479	- 0.015	1.082	- 0.006
Cl	1.525	0.031	1.484	- 0.010	1.084	- 0.004
Br	1.520	0.026	1.485	- 0.009	1.085	- 0.003
I	1.517	0.023	1.486	- 0.008	1.085	- 0.003
Au	1.528	0.034	1.485	- 0.009	1.087	- 0.001

Table S2. Binding energy (kcal/mol) for the κ^1C and $3\kappa^1H$-(TMA)Cl ion pairs in different solvents.

Solvent	κ^1C	$3\kappa^1H$	Diff.
none	-81.30	-99.48	18.18
$CHCl_3$	-78.91	-97.53	18.62
DMF	-76.68	-95.90	19.22
H_2O	-76.49	-95.74	19.25

Table S3. Topological parameters at bond CPs for κ*C* X··· H_3CNMe_3 complexes obtained from QTAIM analysis. ρ is the electron density at the bond CP, $\nabla^2(\rho)$ is the Laplacian electron density of the corresponding bond CP, *H* is the total energy density, G/ρ is the kinetic energy density over ρ ratio.

X	ρ	$\nabla^2(\rho)$
F	0.0379	0.0168
Cl	0.0199	0.0762
Br	0.0171	0.0601
I	0.0145	0.0463
Au	0.0151	0.0494

Table S4. Topological parameters at bond CPs for 3κ*H* X···HCH_2NMe_3 complexes obtained from QTAIM analysis. ρ is the electron density at the bond CP, $\nabla^2(\rho)$ is the Laplacian electron density of the corresponding bond CP, *V* is the electron potential energy density, and $E_{H\cdots X}$ is the energy of the H···X interaction Calculated with the formula $E_{H\cdots X} = 0.5V$.

X	ρ	$\nabla^2(\rho)$
F	0.0347	0.1388
Cl	0.0211	0.0641
Br	0.0183	0.0488
I	0.0154	0.0363
Au	0.0165	0.0395

Table S5. Atomic coordinates of the optimized structures of the κ*C*-X···TMA ion pairs.

κ*C* -(TMA)F

18
kC-(TMA)F

N	-0.673702	-0.000086	-0.000000
C	0.887356	-0.000026	0.000000
H	1.207743	-1.032967	-0.000000
H	1.207634	0.516500	0.894590
C	-1.151869	-0.699840	-1.212002
C	-1.151869	1.399422	0.000000
C	-1.151869	-0.699840	1.212002
H	1.207634	0.516500	-0.894590
H	-0.765736	-0.180466	-2.085806
H	-0.765778	-1.716296	-1.199084
H	-2.241229	-0.709007	-1.228021
H	-2.241226	1.417852	-0.000000
H	-0.765777	1.896449	-0.886720
H	-0.765777	1.896449	0.886720
H	-0.765736	-0.180466	2.085806
H	-2.241229	-0.709007	1.228021
H	-0.765778	-1.716296	1.199084
F	3.091186	0.000339	0.000000

κ*C* -(TMA)Cl

18
kC-(TMA)Cl

N	-0.178349	-1.229351	0.000000
C	0.043644	0.279066	-0.000000
H	1.109191	0.480258	0.000000
H	-0.411725	0.704552	0.887389
C	0.444912	-1.810835	-1.214793
C	-1.636645	-1.504372	-0.000000
C	0.444912	-1.810835	1.214793
H	-0.411725	0.704552	-0.887389
H	-0.013870	-1.356442	-2.089505
H	1.507316	-1.580224	-1.201922
H	0.290123	-2.888494	-1.221235
H	-1.802463	-2.580397	0.000000
H	-2.073216	-1.053215	-0.887654
H	-2.073216	-1.053215	0.887654
H	-0.013870	-1.356442	2.089505
H	0.290123	-2.888494	1.221235
H	1.507316	-1.580224	1.201922
C	0.444912	3.066771	-0.000000

κ*C* -(TMA)Br

18
kC-(TMA)Br

Br	2.482030	-0.012344	-0.000000
N	-2.035905	0.010171	0.000000
C	-0.515657	0.009726	0.000000
H	-0.158532	-1.014845	0.000000
H	-0.158864	0.521947	0.887499
C	-2.521326	-0.691561	-1.215362
C	-2.521326	1.413508	0.000000
C	-2.521326	-0.691561	1.215362
H	-0.158864	0.521947	-0.887499
H	-2.139499	-0.171443	-2.090403
H	-2.139058	-1.709285	-1.202562
H	-3.609913	-0.694478	-1.220047
H	-3.609913	1.419028	0.000000
H	-2.139393	1.911254	-0.887921
H	-2.139393	1.911254	0.887921
H	-2.139499	-0.171443	2.090403
H	-3.609913	-0.694478	1.220047
H	-2.139058	-1.709285	1.202562

κ*C* -(TMA)I

18
kC-(TMA)I

I	-2.623835	-0.047492	0.000000
N	-1.106616	-0.045594	0.000000
C	-0.748546	-1.070445	0.000000
H	-0.749861	0.468197	0.887122
H	-3.109750	-0.749249	-1.215983
C	-3.109750	1.356714	0.000000
C	-3.109750	-0.749249	1.215983
C	-0.749861	0.468197	-0.887122
H	-2.728000	-0.229249	-2.091125
H	-2.729262	-1.767624	-1.203074
H	-4.198203	-0.750849	-1.219809
H	-4.198183	1.360645	0.000000
H	-2.728221	1.854689	-0.887930
H	-2.728221	1.854689	0.887930
H	-2.728000	-0.229249	2.091125
H	-4.198203	-0.750849	1.219809
H	-2.729262	-1.767624	1.203074
H	2.116903	0.038041	-0.000000

κ*C* -(TMA)Au

18
kC-(TMA)Au

Au	1.616168	0.000016	-0.000010
N	-2.987377	0.000045	-0.000050
C	-1.459870	0.004931	0.004251
H	-1.102528	-0.450384	0.923975
H	-1.095616	-0.561264	-0.848726
C	-3.477425	0.776565	1.167002
H	-3.099114	1.792892	1.089852
H	-4.566235	0.776022	1.169218
C	-3.470228	0.621074	-1.259555
H	-4.559020	0.618890	-1.268395
H	-3.092857	1.639405	-1.309963
C	-3.466728	-1.402745	0.088338
H	-3.080279	-1.954676	-0.764923
H	-3.085936	-1.841383	1.007291
H	-3.083068	0.048297	-2.098628
H	-4.555507	-1.412792	0.085647
H	-3.096360	0.314882	2.074536
H	-1.103578	1.029600	-0.058978

Table S6. Atomic coordinates of the optimized structures of the 3κ*H*-X···TMA ion pairs.

3κ*H* -(TMA)F

18
3kH-(TMA)F

N	-0.170824	0.486870	-0.000000
C	1.327093	0.457830	0.000000
H	1.593427	-0.610659	0.000000
H	1.672324	0.977325	0.892804
C	-0.659948	-0.238792	-1.215905
C	-0.659948	1.881966	-0.000000
C	-0.659948	-0.238792	1.215905
H	1.672324	0.977325	-0.892804
H	-0.289565	0.289375	-2.093265
H	-0.249134	-1.256351	-1.127630
H	-1.748726	-0.222290	-1.200871
H	-1.748143	1.876651	-0.000000
H	-0.292018	2.387260	-0.890887
H	-0.292018	2.387260	0.890887
H	-0.289565	0.289375	2.093265
H	-1.748726	-0.222290	1.200871
H	-0.249134	-1.256351	1.127630
F	0.786803	-2.244222	0.000000

3κ*H* -(TMA)Cl

18
3kH-(TMA)Cl

N	-0.334345	0.945123	0.000000
C	1.162668	0.934958	0.000000
H	1.479851	-0.113386	-0.000000
H	1.502777	1.455971	0.893096
C	-0.828355	0.230842	-1.219295
C	-0.828355	2.341890	0.000000
C	-0.828355	0.230842	1.219295
H	1.502777	1.455971	-0.893096
H	-0.458403	0.762665	-2.093952
H	-0.439505	-0.792195	-1.175919
H	-1.916547	0.246425	-1.200888
H	-1.916296	2.332018	0.000000
H	-0.461103	2.846564	-0.891131
H	-0.461103	2.846564	0.891131
H	-0.458403	0.762665	2.093952
H	-1.916547	0.246425	1.200888
H	-0.439505	-0.792195	1.175919
Cl	0.838636	-2.370855	-0.000000

3κ*H* -(TMA)Br

18
3kH-(TMA)Br

Br	2.047752	-0.001104	0.000000
N	-1.673768	0.000959	0.000000
C	-1.167001	1.409083	0.000000
H	-0.073472	1.367051	0.000000
H	-1.543983	1.903652	0.893258
C	-1.167001	-0.703346	-1.219460
C	-3.156173	0.001069	0.000000
C	-1.167001	-0.703346	1.219460
H	-1.543983	1.903652	-0.893258
H	-1.542800	-0.176349	-2.094456
H	-0.073587	-0.683261	-1.182284
H	-1.545301	-1.723663	-1.201635
H	-3.509151	-1.027972	0.000000
H	-3.508957	0.515656	-0.891255
H	-3.508957	0.515656	0.891255
H	-1.542800	-0.176349	2.094456
H	-1.545301	-1.723663	1.201635
BH	-0.073587	-0.683261	1.182284

3κ*H* -(TMA)I

18
3kH-(TMA)I

N	-1.785694	1.281932	-0.000000
C	-0.554249	2.131592	-0.000000
H	0.313326	1.466032	-0.000000
H	-0.570926	2.752732	0.893424
C	-1.785694	0.415155	-1.219729
C	-2.990700	2.146528	-0.000000
C	-1.785694	0.415155	1.219729
H	-0.570926	2.752732	-0.893424
H	-1.784138	1.061801	-2.094974
H	-0.887511	-0.207606	-1.189334
H	-2.686208	-0.195506	-1.201478
H	-3.877053	1.515769	-0.000000
H	-2.977072	2.770170	-0.891291
H	-2.977072	2.770170	0.891291
H	-1.784138	1.061801	2.094974
H	-2.686208	-0.195506	1.201478
H	-0.887511	-0.207606	1.189334
I	1.444779	-1.037152	0.000000

3κ*H* -(TMA)Au

18
3kH-(TMA)Au

Au	1.342190	-0.000001	0.000000
N	-2.474875	0.000012	0.000008
C	-1.966452	0.757586	-1.185398
H	-2.338647	1.778400	-1.121662
H	-0.870350	0.733793	-1.147882
C	-3.957692	0.000126	0.000269
H	-4.309765	-0.473758	-0.913674
H	-4.309416	-0.554472	0.867743
C	-1.966449	-1.405376	-0.063559
H	-2.338097	-1.941105	0.807941
H	-2.338967	-1.860586	-0.979337
C	-1.966011	0.647661	1.248685
H	-0.869903	0.627025	1.208880
H	-2.337937	1.670159	1.277173
H	-0.870326	-1.360954	-0.062088
H	-2.337933	0.082020	2.100961
H	-4.309492	1.028650	0.046952
H	-2.338390	0.270848	-2.085059

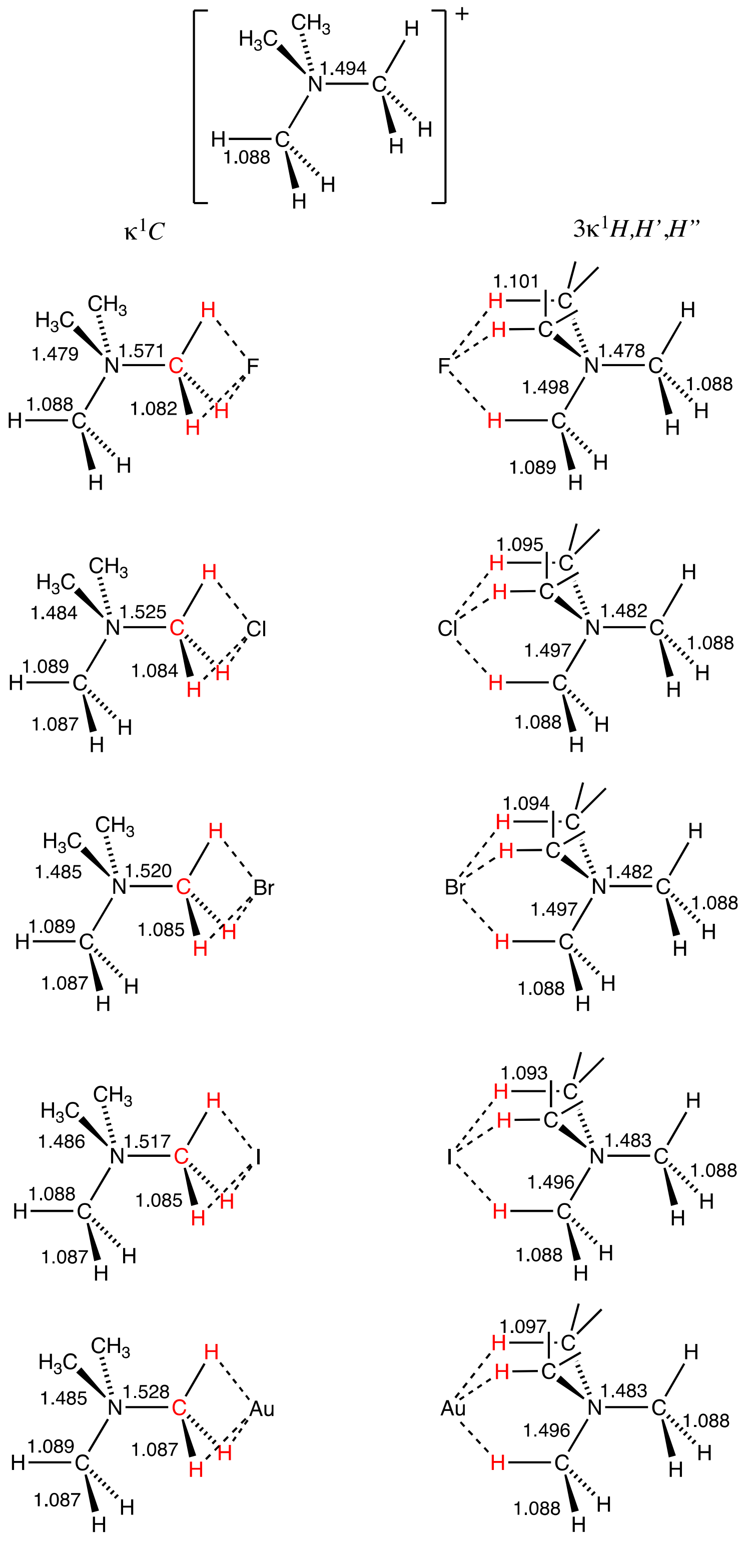

Figure S1. Optimized bond distances (in Å) of the independent TMA cation (first row) and in the X···TMA ion pairs (X = F, Cl, Br, I and Au) with the two alternative energy minima, κ[1]*C* and 3 κ[1]*H*, shown with the cation in the same orientation to facilitate comparison.

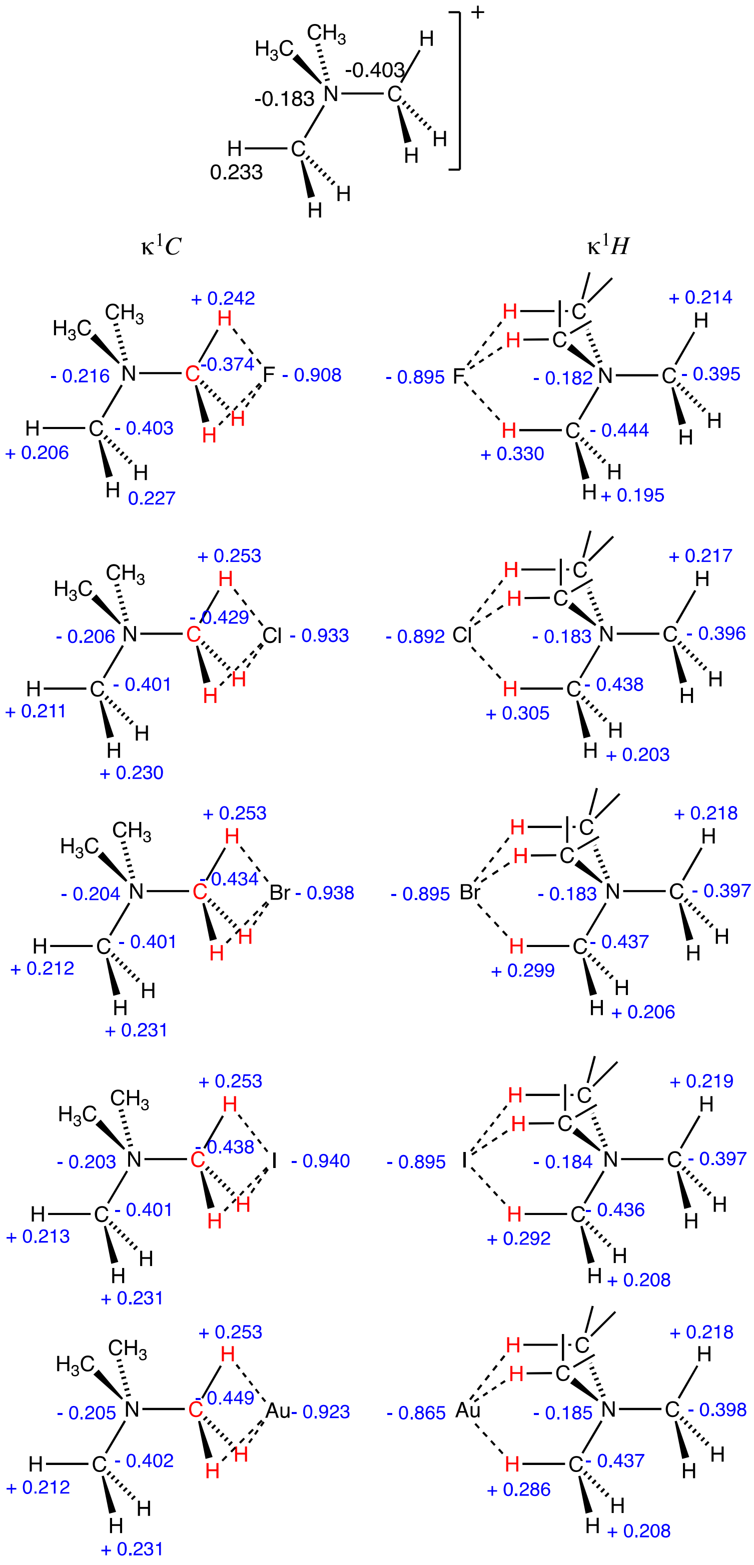

Figure S2. NPA atomic charges calculated for the X···TMA ion pairs in the two energy minima with effective C_{3v} symmetry.

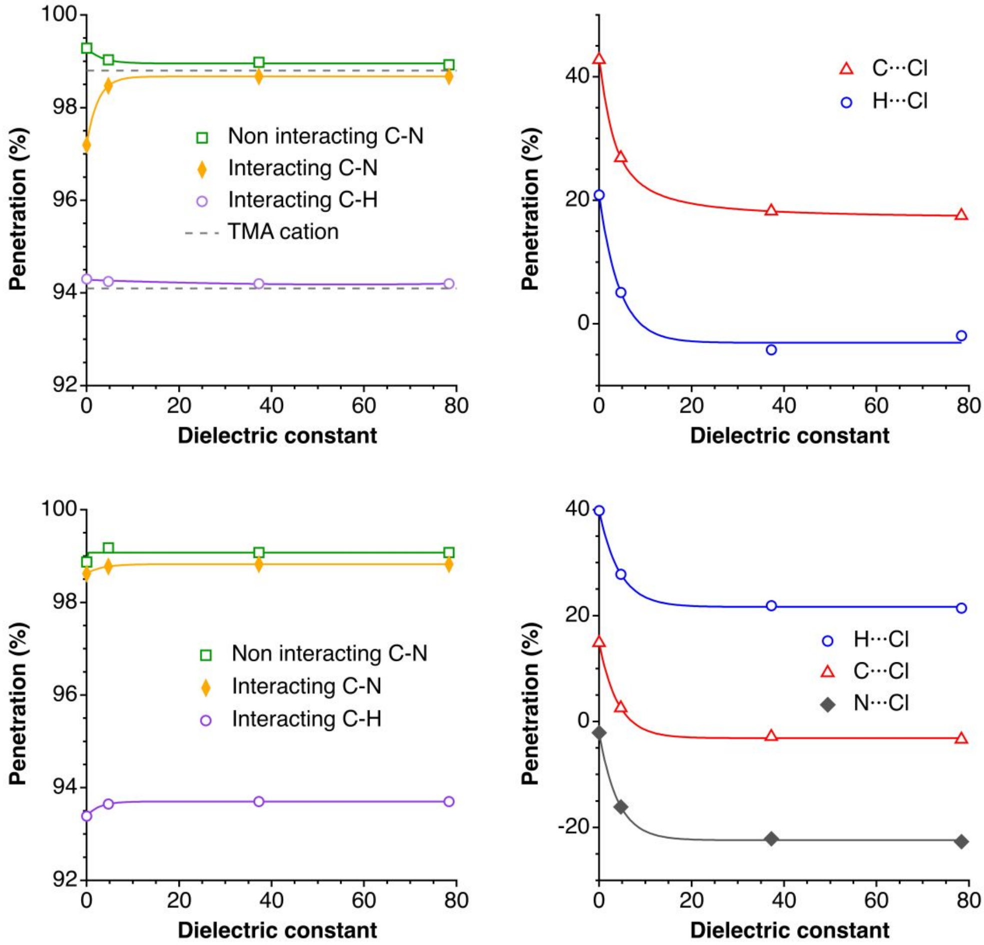

Figure S3. Effect of the solvent (PCM calculations) on the C-N and C-H bond distances within the TMA cation (left) and on the Cl ···E contacts (E = H, C, N; right) of the Cl···TMA ion pair with the κ^1-*C* (above) and $3\kappa^1$-*H* (below) interaction modes.
